# circTADA2As suppress breast cancer progression and metastasis via targeting miR-203a-3p/SOCS3 axis

**DOI:** 10.1038/s41419-019-1382-y

**Published:** 2019-02-20

**Authors:** Jian-Zhen Xu, Chang-Chun Shao, Xiao-Jia Wang, Xing Zhao, Jun-Qing Chen, Yan-Xiu Ouyang, Jun Feng, Fan Zhang, Wen-He Huang, Qian Ying, Chun-Fa Chen, Xiao-Long Wei, Hong-Yan Dong, Guo-Jun Zhang, Min Chen

**Affiliations:** 1 Department of Bioinformatics, Shantou University Medical College (SUMC), 515041 Shantou, China; 2 ChangJiang Scholar’s Laboratory, Shantou University Medical College, 515041 Shantou, China; 3 Key Lab of Diagnosis & Treatment Technology on Thoracic Oncology, Zhejiang Cancer Hospital, 310000 Hangzhou, China; 4 Guangdong Provincial Key Laboratory on Breast Cancer Diagnosis and Treatment, Cancer Hospital of Shantou University Medical College, 515041 Shantou, China; 5 The Breast Center, Cancer Hospital of Shantou University Medical College, 515041 Shantou, China; 6 Department of Thyroid and Breast Surgery, First Affiliated Hospital of Shantou University Medical College, 515041 Shantou, China; 7 Department of Pathology, Cancer Hospital of Shantou University Medical College, 515041 Shantou, China; 8 Department of Pathology, Linyi People’s Hospital, 276000 Linyi, China; 9 The Cancer Center, Xiang’an Hospital of Xiamen University, 2000 Xiang’an East Rd., 361111 Xiamen, Fujian China

## Abstract

More and more evidence indicates that circular RNAs (circRNAs) have important roles in several diseases, especially in cancers. However, their involvement remains to be investigated in breast cancer. Through screening circRNA profile, we identified 235 differentially expressed circRNAs in breast cancer. Subsequently, we explored the clinical significance of two circTADA2As in a large cohort of triple-negative breast cancer (TNBC), and performed functional analysis of circTADA2A-E6 in vitro and in vivo to support clinical findings. Finally, we evaluated the effect of circTADA2A-E6 on miR-203a-3p and its target gene *SOCS3*. We detected two circRNAs, circTADA2A-E6 and circTADA2A-E5/E6, which were among the top five differentially expressed circRNAs in breast cancer. They were consistently and significantly decreased in a large cohort of breast cancer patients, and their downregulation was associated with poor patient survival for TNBC. Especially, circTADA2A-E6 suppressed in vitro cell proliferation, migration, invasion, and clonogenicity and possessed tumor-suppressor capability. circTADA2A-E6 preferentially acted as a miR-203a-3p sponge to restore the expression of miRNA target gene *SOCS3*, resulting in a less aggressive oncogenic phenotype. circTADA2As as promising prognostic biomarkers in TNBC patients, and therapeutic targeting of circTADA2As/miRNA/mRNA network may be a potential strategy for the treatment of breast cancer.

## Introduction

Breast cancer is the most frequently occurring cancer, ranking no. 1 in women worldwide^1^. Breast cancer is a complex and heterogeneous disease characterized by different molecular alterations^2^. At least five independent intrinsic molecular subtypes [luminal A (LA), luminal B (LB), Her-2 overexpressed, triple-negative breast cancer (TNBC), and normal breast-like] have been consistently reported in different cohorts^3,4^. Recently, multiple gene chip tests for breast cancer biomarkers have been used for assessing prognosis and chemo-strategies, such as the 21-gene signature (Oncotype DX)^5^ and the 70-gene prognosis-signature MammaPrint^6^. Despite intensive investigations, therapeutic improvements, and prolonged survival over the last few decades, many patients with breast cancer cannot escape eventual recurrence, metastasis, and chemo-resistance. This highlights the need to unravel yet unknown biomarkers as well as the underlying mechanisms for breast cancer malignancy.

With the rapid development of deep sequencing and microarray technologies in recent years, increasing evidence confirms that the mammalian genome encodes thousands of circular RNAs (circRNAs)^7,8^. These RNAs are characterized as a class of RNA molecules with a circular configuration formed by either typical spliceosome-mediated or lariat-type splicing between an upstream splice acceptor and a downstream splice donor^9^. circRNAs have high stability and sequence conservation in mammalian cells with tissue and developmental stage-specific features^10–12^. circRNAs arise in exonic, intronic, and intergenic regions^13^, and regulate gene expression by acting as a scaffold in the assembly of protein complexes^14^, modulating the expression of parental genes^15^, regulating alternative splicing^16^ and RNA-protein interactions^17,18^, and sponging miRNAs^19,20^. Thus, it is worth exploring circRNAs for functional relevance in breast cancer.

Besides having the potential to regulate cellular processes, studies have reported that circRNAs are associated with human diseases^21,22^. Recently, circRNAs have been shown to be potential novel diagnostic^23,24^ and prognostic biomarkers for cancers^25,26^. circRNAs are present in many human body fluids, such as plasma^27^ and saliva^28^, and have also been detected in exosomes^29^. Thus, the crucial roles and functions of circRNA are becoming a novel focus in cancer research^30,31^. circRNA microarrays and RNA sequencing in tumor tissues have been used to explore cancer-related differentially expressed circRNAs in various types of cancer, e.g., esophageal carcinoma^15^, hepatocellular carcinoma^23^, gastric cancer^32^, and breast cancer^33^. By analyzing high-throughput transcriptome sequencing data from a large cohort of breast cancer patients in the Cancer Genome Atlas (TCGA) consortium, Nair et al.^34^ identified novel circRNAs specific for breast cancer, and cataloged those unique circRNAs to different breast cancer subtypes, suggesting that unique circRNAs can be used to distinguish breast cancer subtypes. However, there is a lack of experimental and clinical evidence for the roles of circRNAs in breast cancer. The identification of unique features of circRNAs in breast cancer requires more intensive investigation of breast carcinogenesis and disease progression.

In the present study, we identified differentially expressed circRNAs in breast cancer. Subsequently, we investigated the clinical significance of two highly expressed circTADA2As (TADA2A is a gene, named Transcriptional Adaptor 2A), circTADA2A-E6 and circTADA2A-E5/E6 in a larger cohort of TNBC patients. We have made attempts to fill the gap in knowledge for the molecular contribution of circTADA2A-E6 in breast cancer pathogenesis by performing a systematic and comprehensive functional analysis and by exploring its regulatory effect on gene expression.

## Results

### Differential expression of circRNAs in breast cancer

**Fig. 1:**
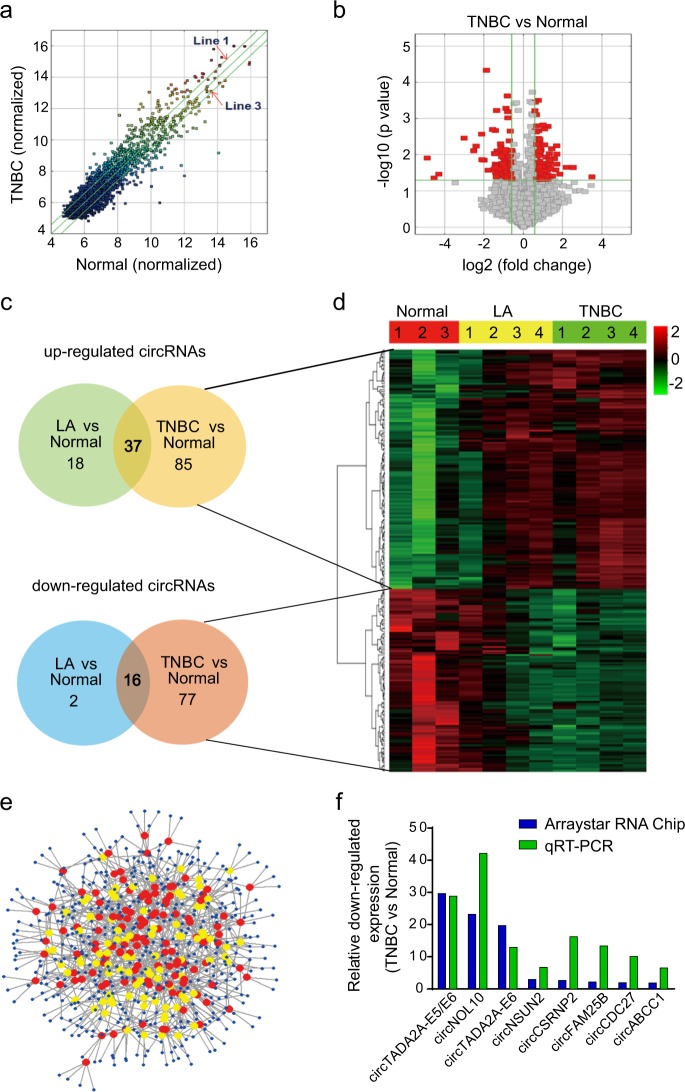
Profiling of circular RNAs (circRNAs) from breast cancer patients’ specimens. **a** Scatter plot. Green lines represent fold-change. Above line 1 and below line 3 indicate >1.5-fold-change in circRNA expression level in TNBC (*N* = 4) as compared to NMGT (*N* = 3). **b** Volcano plot. The vertical green lines correspond to 1.5-fold upregulated and downregulation, and the horizontal green line represents *p* value of 0.05. The red points in the plot represent circRNAs with statistically significant differential expression. **c** circRNAs profile for TNBC and LA (*N* = 4) subtypes as compared to NMGT. **d** Clustered heatmap for differentially expressed circRNAs. Rows represent circRNAs and columns represent tissue types. circRNAs were classified according to the Pearson correlation. The color scale runs from green (low intensity) to black (medium intensity), to red (strong intensity). **e** Network of 215 differentially expressed circRNAs and the predicted target miRNAs in TNBC. Nodes are represented in different colors: red for upregulated circRNAs, yellow for downregulated circRNAs, and blue for predicted miRNA sponge circRNAs. **f** Relative downregulated expression of circRNAs by microarray data and quantitative reverse transcription polymerase reaction (qRT-PCR) in TNBC

To identify differentially expressed circRNAs in breast cancer, we performed high-throughput circRNA microarray analysis and generated circRNA expression profiles from eight patients’ specimens (TNBC, *N* = 4; luminal A, *N* = 4) and three normal mammary gland tissues (NMGT) (Supplementary Table 1 and Supplementary Figure S1). Compared to NMGT, scatter and volcano plot filtering identified differentially expressed circRNAs in breast cancer specimens (Fig. 1a, b). 140 upregulated and 95 downregulated circRNA transcripts were identified, including 215 and 73 circRNAs for the TNBC and LA subtypes, respectively (Fig. 1c). The majority of the differentially expressed circRNAs were spliced from gene exons (Supplementary Table 2) data for which are presented as distinct expression clusters (Fig. 1d). Four hundred sixty-five miRNAs were predicted to bind to 215 circRNAs (Fig. 1e) in TNBC. Collectively, these data demonstrated the presence of a wide array of differentially expressed circRNAs in breast cancer. To validate microarray data, we selected eight differentially expressed circRNAs in TNBC (Supplementary Table 3) and confirmed their dysregulation by quantitative reverse transcription polymerase reaction (qRT-PCR) (Fig. 1f). Among these circRNAs, the expression of two circTADA2As was consistently and significantly decreased in TNBC.

### Downregulation of two circTADA2As in breast cancer subtypes and cell lines

**Fig. 2:**
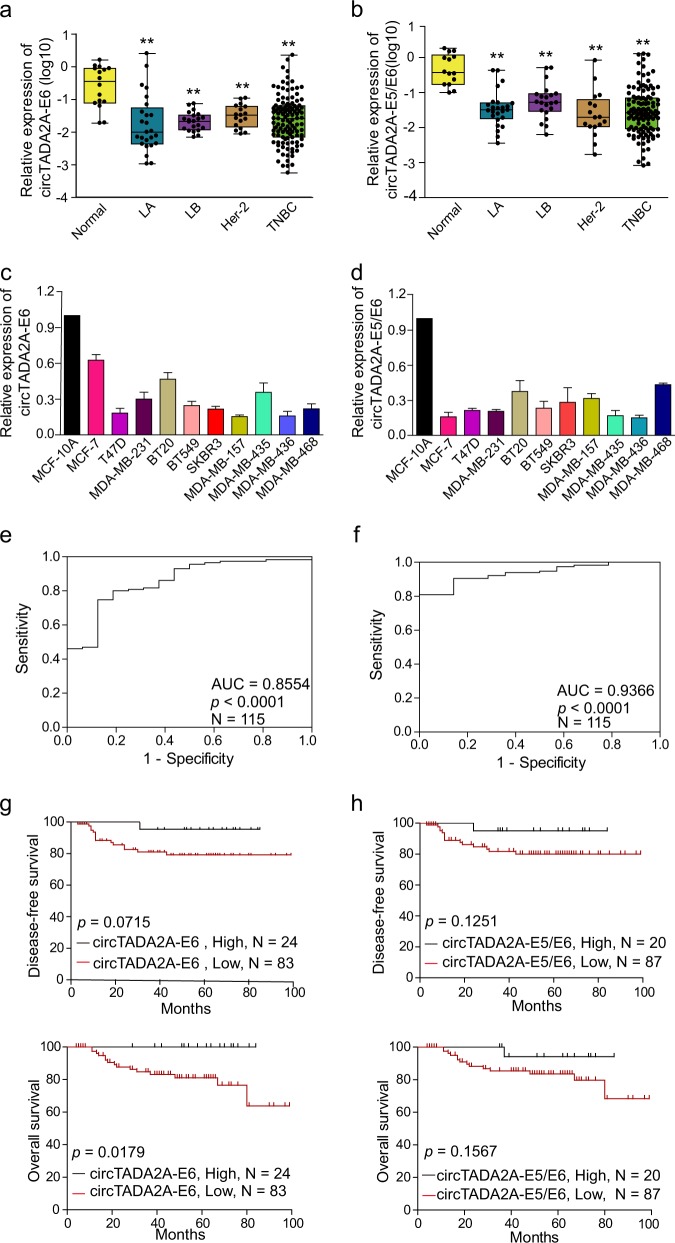
Decreased expression of two circTADA2As in breast cancer and their clinical implications. **a**, **b** Relative expression levels of circTADA2A-E6 (**a**) and circTADA2A-E5/E6 (**b**) were analyzed by quantitative reverse transcription polymerase reaction (qRT-PCR) in breast cancer subtypes (LA, *N* = 25; LB, *N* = 21; Her-2, *N* = 17; and TNBC, *N* = 115) as compared to NMGT tissues (*N* = 16). Middle horizontal lines in the scatter plot represent the median. *p < 0.05, ** *p* < 0.01. **c**, **d** The expression levels of circTADA2A-E6 (**c**) and circTADA2A-E5/E6 (**d**) were analyzed by qRT-PCR in 10 breast cancer cell lines and an immortalized mammary gland cell line, MCF-10A. **e**, **f** Receiver operating characteristic (ROC) analysis for circTADA2A-E6 (**e**) and circTADA2A-E5/E6 (**f**) in TNBC using NMGT as a control. **g**, **h** Kaplan–Meier survival curve analysis for the correlation between circTADA2A-E6 (**g**) and circTADA2A-E5/E6 (**h**) expression and disease-free survival (DFS) or overall survival (OS) in TNBC

We focused on two circTADA2As, which were ranked in the top five downregulated circRNAs by microarray analysis, and validated their expression levels by qRT-PCR. We found that circTADA2A-E6 (hsa_circ_0006220) and circTADA2A-E5/E6 (hsa_circ_0043278), were spliced from exon 6 or exons 5 and 6 of the same TADA2A gene (chr17, 35766977–35839830), respectively. Their expression levels were analyzed in different breast cancer subtypes (*N* = 178) and ten breast cancer cell lines. As shown in Fig. 2a, b, expression of each of the two circTADA2As was markedly decreased in different breast cancer subtypes (3.979- to 28.869-fold, respectively; both *p* < 0.01). Similarly, the expression levels of these two circTADA2As in ten breast cancer cell lines were lower (1.6- to 6.5-fold) than in the immortalized mammary gland cell line MCF-10A (Fig. 2c, d). Thus, these data confirmed the downregulation of the two circTADA2As in breast cancer.

### Clinical implications of decreased circTADA2A expression in TNBC

To address the potential clinical implications of the two downregulated circTADA2As in breast cancer, we analyzed the relationship between these two circRNAs and clinicopathological features of patients with TNBC (*N* = 115). As shown in Table 1, decreased circTADA2A-E6 expression was significantly associated with increased lymphatic metastasis (*p* *=* 0.012) and advanced clinical stage (*p* *=* 0.022), but not with age or T classification. However, no association was found between decreased circTADA2A-E5/E6 expression and any of the clinicopathological parameters (Supplementary Table 4). Interestingly, the area under the curve (AUC) was 0.8554 for circTADA2A-E6 (*p* *<* 0.0001, Fig. 2e) and 0.9366 for circTADA2A-E5/E6 (*p* *<* 0.001, Fig. 2f), which indicates that 85.5% (98/115) and 93.7% (106/115) of TNBC patients had lower expression levels of circTADA2A-E6 and circTADA2A-E5/E6, respectively, than healthy individuals, implying that each circTADA2A might have a potential diagnostic value. More importantly, patients with lower levels of circTADA2A-E6 had poor prognosis as evidenced by shorter disease-free survival (DFS) (*p* *=* 0.0715, Fig. 2g, upper panel) and overall survival (OS) (*p* *=* 0.0179, Fig. 2g, bottom panel). Low expression levels of circTADA2A-E5/E6 were also associated with shorter DFS and OS, but these results did not achieve significance (*p* > 0.05, Fig. 2h). We performed univariate and multivariate analyses (UA and MA) to determine the relationships between DFS or OS and circTADA2A-E6 or circTADA2A-E5/E6 expression levels along with four clinical parameters: age, TNM stage, T classification, and lymphatic metastasis. As shown in Table 2, except for the associations between lymphatic metastasis and T classification status and DFS or OS, patients expressing mid-range levels of circTADA2A-E6 were specifically associated with poor DFS (UA, hazard ratio (HR) = 10.585, 95% confidence interval (CI) = 1.299–86.267, *p* *=* 0.028) and OS (UA, HR = 11.426, 95% CI = 1.4–93.227, *p* *=* 0.023; MA, HR = 8.365, 95% CI = 0.938–74.599, *p* *=* 0.057). Nonetheless, no significant correlation was detected between DFS or OS and the expression level of circTADA2A-E5/E6. Thus, these results indicated that circTADA2As with potential tumor-suppressor characteristics might be a new biomarker for breast cancer.

**Table 1:** The correlation between clinicopathological factors and circTADA2A-E6 expression (2^−ΔΔCt^) in TNBC

Characteristic	No. of patients (%)	Mean ± SEM	*p* value
Age			
≥50	67 (58.3%)	0.11374 ± 0.04272	0.548
<50	48 (41.7%)	0.08113 ± 0.02307	
AJCC TNM stage^a^			
I–II	82 (74.5%)	0.10260 ± 0.03167	0.022*
III–IV	28 (25.5%)	0.02734 ± 0.00592	
T classification^b^			
T_1_	25 (24.0%)	0.05275 ± 0.02148	0.697
T_2_	70 (67.3%)	0.10640 ± 0.03645	
T_3_	5 (4.8%)	0.02967 ± 0.01206	
T_4_	4 (3.8%)	0.00879 ± 0.00385	
Lymphatic metastasis			
N_0–1_	91 (79.1%)	0.11838 ± 0.03339	0.012*
N_2–3_	24 (20.9%)	0.03093 ± 0.00663	

*AJCC* American Joint Committee on Cancer

*p < 0.05

^a^4.35% patient information missing

^b^9.56% patient information missing

**Table 2:** Univariate and multivariate analysis of the relationship between circTADA2As expression (2^−ΔΔCt^) and DFS or OS in TNBC

Variable	DFS							OS						
	Samples	Univariate analysis			Multivariate analysis (*N* = 99)			Samples	Univariate analysis			Multivariate analysis (*N* = 99)		
		HR	95% CI	*p* value	HR	95% CI	*p* value		HR	95% CI	*p* value	HR	95% CI	*p* value
Age (<50 /≥50)	107	0.81	0.304–2.157	0.673				107	1.291	0.459–3.634	0.629			
AJCC TNM stage (III–IV/I–II)^a^	104	6.188	2.243–17.07	0.000**				104	5.301	1.882–14.928	0.002**			
T classification (T_3–4_/T_1–2_)^b^	99	3.726	1.037–13.384	0.044*	4.153	1.125–15.335	0.033*	99	6.616	1.987–22.032	0.002**	11.515	2.908–45.593	0.001**
Lymphatic metastasis (N_2–3_/N_0–1_)	107	6.627	2.458–17.866	0.000**	5.261	1.817–15.228	0.002**	107	5.640	2.038–15.610	0.001**	4.94	1.531–15.916	0.008**
circTADA2A-E6	107			0.031*				107			0.018*			0.057*
High	25							25						
Medium	21	10.585	1.299–86.267	0.028*				21	11.426	1.4–93.227	0.023*	8.365	0.938–74.599	0.057
Low	61	3.811	0.476–30.481	0.207				61	3.497	0.429–28.486	0.106	2.34	0.279–19.644	0.434
circTADA2A-E5/E6	107			0.424				107			0.394			
High	16							16						
Medium	31	2.437	0.272–21.823	0.426				32	3.877	0.465–32.330	0.211			
Low	60	3.575	0.461–27.715	0.223				59	2.372	0.295–19.065	0.416			

*AJCC* American Joint Committee on Cancer

*p < 0.05

**p < 0.01

^a^2.8% patient information missing

^b^7.48% patient information missing

### circTADA2A-E6 suppresses cancer progression

**Fig. 3:**
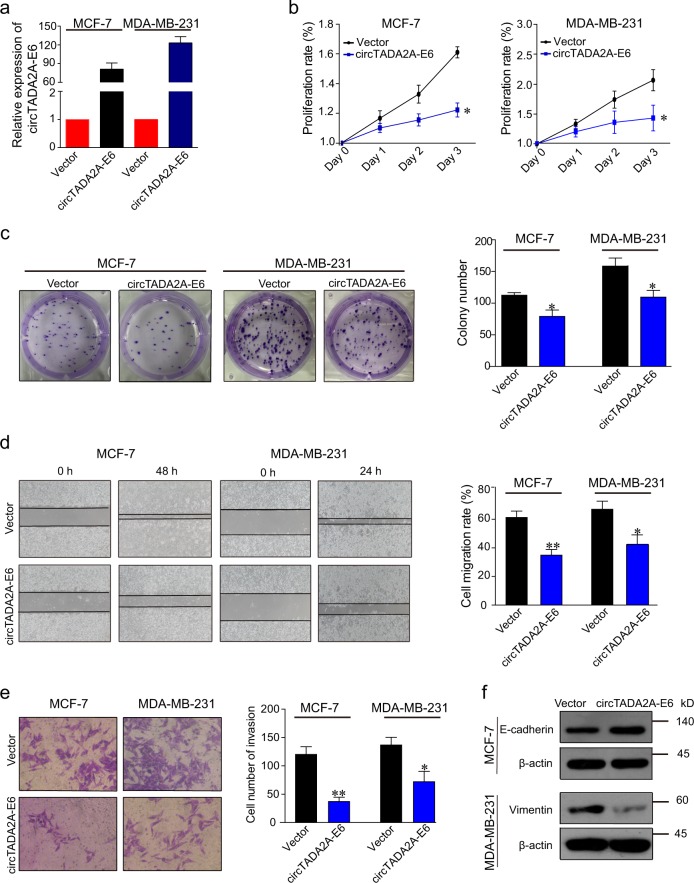
circTADA2A-E6 suppresses cancer progression. **a**–**f** Forty-eight hours after transfecting MCF-7 and MDA-MB-231 cells with a vector expressing circTADA2A-E6, ectopic circTADA2A-E6 expression was analyzed by quantitative reverse transcription polymerase reaction (qRT-PCR) (**a**). The effect of circTADA2A-E6 on cell viability was analyzed by CCK-8 (**b**). The effect of circTADA2A-E6 on colony formation after two- to three-week transfection was determined via a clonogenicity assay (left, representative pictures; right, quantitative bar for colony numbers) (**c**). The effect of circTADA2A-E6 on cell migration was determined via wound scratch assay (left, representative images; quantitative bar for cell migration rate) (**d**). The effect of circTADA2A-E6 on cell invasion was determined via Transwell assay (left, morphological comparison of cell penetration; quantitative bar for number of penetrating cells) (**e**). The effect of circTADA2A-E6 on the expression levels of E-cadherin and vimentin was analyzed by western blotting (WB) (**f**). Error bars represent the mean ± SEM from three independent experiments. *p < 0.05, **p < 0.01

To investigate the role of circTADA2A-E6 in breast cancer, we overexpressed circTADA2A-E6 in breast cancer cell lines. Successful ectopic circTADA2A-E6 expression (74.91- and 132.79-fold increase in MCF-7 and MDA-MB-231 cells, respectively, Fig. 3a) decreased cell proliferation (Fig. 3b), inhibited colony formation by 30.09 (34/113) or 30.81% (49/159, Fig. 3c), tumor cell migration by 26.36 or 24.69% (Fig. 3d), cell invasion by 69.17 (83/120) or 47.44% (65/137, Fig. 3e) in MCF-7 or MDA-MB-231 cells, respectively. Also, ectopic circTADA2A-E6 expression increased E-cadherin expression in MCF-7 cells, but decreased vimentin expression in MDA-MB-231 cells (Fig. 3f).

**Fig. 4:**
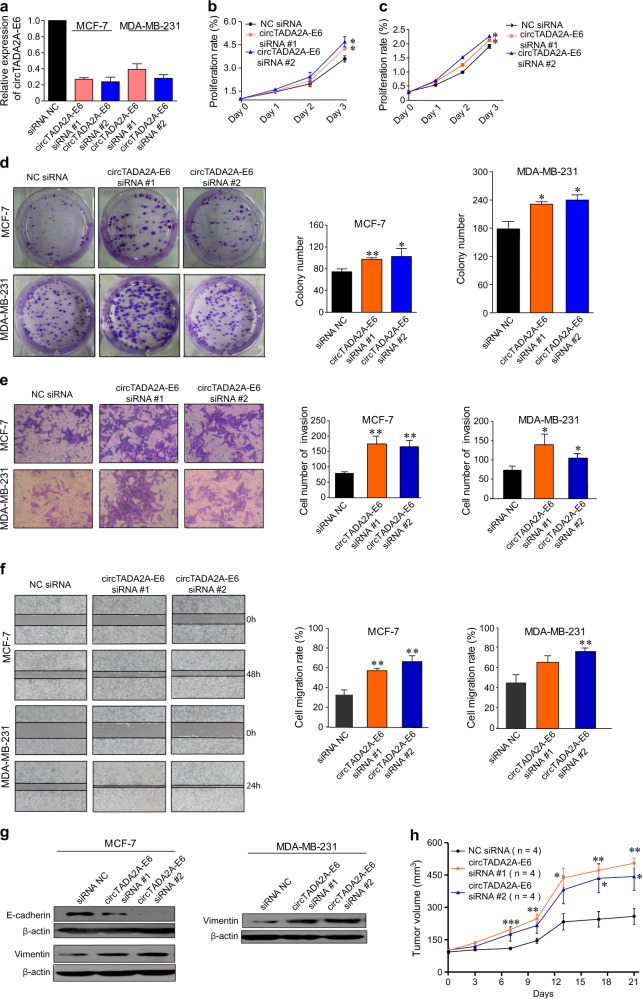
Knockdown of circTADA2A-E6 promotes cellular proliferation, clonogenicity, migration, and invasion. **a**–**g** After transfecting MCF-7 and MDA-MB-231 cells for 48 h with circTADA2A-E6 siRNA #1 and #2, circTADA2A-E6 expression was analyzed by quantitative reverse transcription polymerase reaction (qRT-PCR) (**a**). The effect of circTADA2A-E6 siRNAs on cell viability was analyzed by CCK-8 (**b** for MCF-7 and **c** for MDA-MB-231); the effect of circTADA2A-E6 siRNA on colony formation after two- to three-week transfection was determined via clonogenicity assay (left, representative pictures; right, quantitative bar for colony numbers) (**d**). The effect of circTADA2A-E6 siRNA on cell migration was determined via wound scratch assay (left, representative images; quantitative bar for cell migration rate) (**e**). The effect of circTADA2A-E6 siRNA on cell invasion was determined via Transwell assay (left, morphological comparison of cell penetration; quantitative bar for number of penetrating cells) (**f**). The effect of circTADA2A-E6 siRNA on the expression levels of E-cadherin and vimentin was analyzed via western blotting (WB) (**g**). **h** The tumor growth curve at the time the animals were sacrificed in different treatment groups. Error bars represent the mean ± SEM from three independent experiments. *p < 0.05, **p < 0.01

To further confirm the anti-tumor activity of circTADA2A-E6, we used RNA interference to knockdown its expression in breast cancer cell lines. Successful circTADA2A-E6 knockdown (73 and 76% knockdown for circTADA2A-E6 siRNA #1 and #2, respectively, in MCF-7; 61 and 72% knockdown for circTADA2A-E6 siRNA #1 and #2, respectively in MDA-MB-231, Fig. 4a) increased cell growth (Fig. 4b, c), promoted colony formation (1.3- and 1.38-fold for siRNA #1 and #2 in MCF-7, respectively; 1.3- and 1.34-fold siRNA #1 and #2 in MDA-MB-231, respectively, *p* *<* 0.05, Fig. 4d), increased cell invasion (2.2- and 2.1-fold for siRNA #1 and #2 in MCF-7, respectively; 1.9- and 1.4-fold siRNA #1 and #2 in MDA-MB-231, respectively, Fig. 4e), and cell migration (1.6- and 1.9-fold for siRNA #1 and #2 in MCF-7, respectively; 1.5- and 1.8-fold for siRNA #1 and #2 in MDA-MB-231, respectively, Fig. 4f). Furthermore, silencing circTADA2A-E6 significantly decreased E-cadherin expression in MCF-7 cells and increased Vimentin expression in MDA-MB-231 cells (Fig. 4g). We further assessed whether circTADA2A-E6 siRNAs promoted tumor growth in mouse xenografts. As illustrated in Fig. 4h and Supplementary Figure S2, the tumor nodules in nude mice injected with circTADA2A-E6 siRNAs grew significantly faster compared to the controls. Thus, these gain-of-function and loss-of-function studies suggested that circTADA2A-E6 has a role in breast cancer progression and metastasis and can serve as a potential therapeutic target.

### circTADA2A-E6 acts as a sponge for miRNAs and restores gene expression

**Fig. 5:**
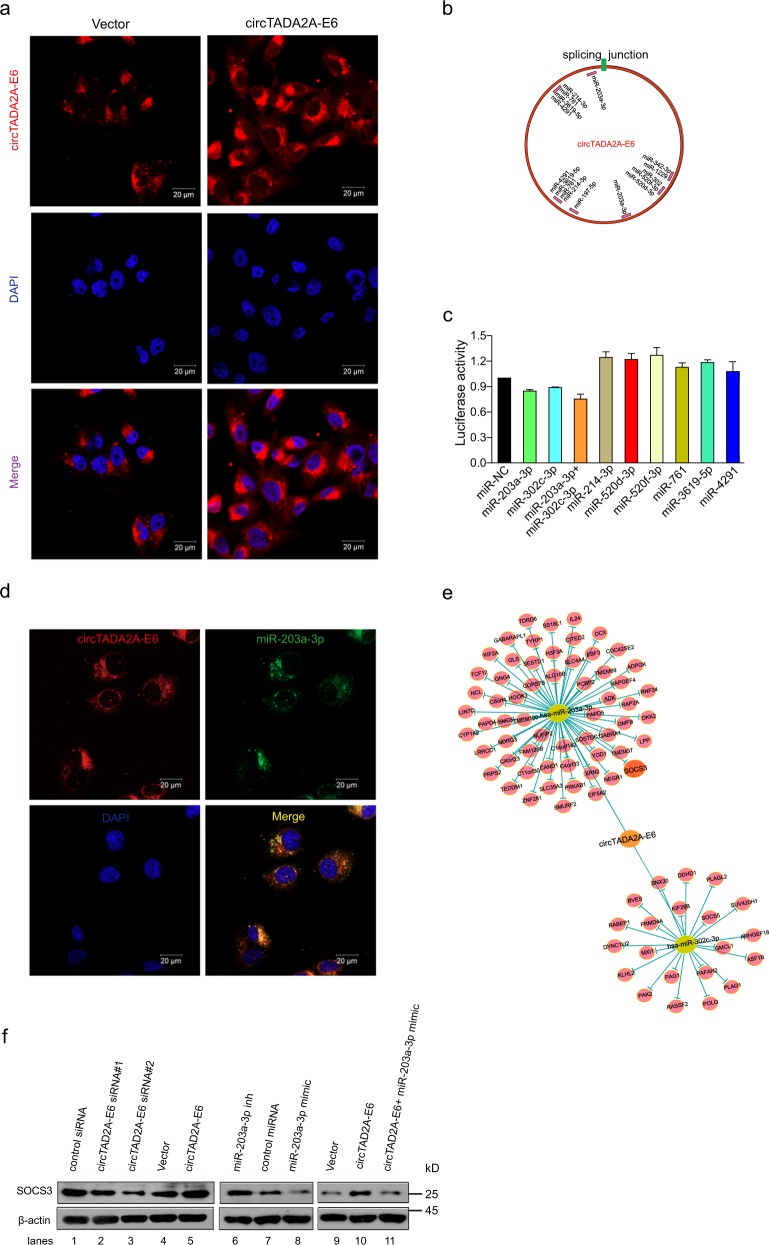
circTADA2A-E6 binds to miR-203a-3p and restores the expression of *SOCS3*. **a** After circTADA2A-E6 or empty vectors were transfected in MDA-MB-231 cells, the localization of endogenous or ectopic circTADA2A-E6 was detected by RNA in situ hybridization (FISH). Red, Cy3-labeled probes specific to circTADA2A-E6; blue, DAPI stain for nuclei. Scale bar, 20 μm. **b** A schematic drawing showing the putative binding sites of the miRNAs associated with circTADA2A-E6. miR-214 family members (miR-214-3p, miR-761, miR-3619-5p, and miR-4291) share the same binding site as miR-302 family members (miR-302c-3p, miR-520d-3p, and miR-520f-3p) and miR-342 family members (miR-342-3p and miR-1229). **c** After co-transfecting phRluc-circTADA2A-E6 plasmids with miRNA mimics in 293T cells, *Renilla* luciferase activity was measured. **d** FISH analysis on the co-localization of circTADA2A-E6 and miR-203a-3p in MCF-7 cells. Red, Cy3-labeled probes specific to circTADA2A-E6; green, FITC-labeled probes specific to miR-203a-3p; blue, DAPI stain for nuclei. Scale bar, 20 μm. **e** The circTADA2A-E6/miR-203a-3p and miR-302c-3p/mRNA axes were generated after Cytoscape analysis. **f** After transfecting MCF-7 cells with the circTADA2A-E6 vector, siRNA #1 or #2, miR-203a-3p mimic, or miR-203a-3p inhibitor, the expression of *SOCS3* was analyzed by western blotting (WB)

**Fig. 6:**
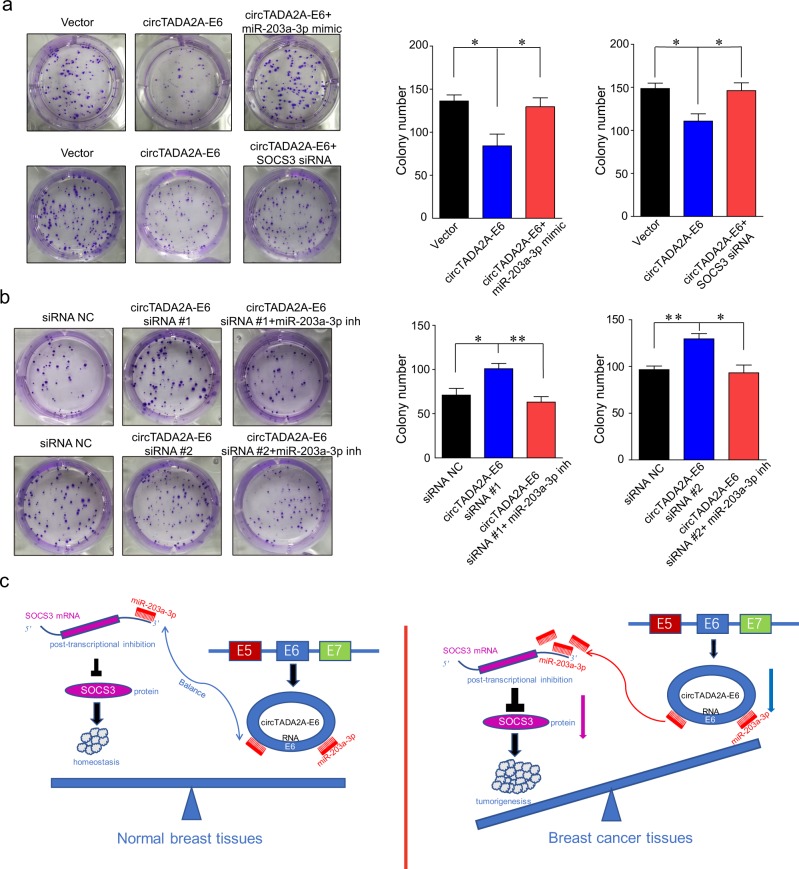
The circTADA2A-E6/miR-203a-3p/*SOCS3* axis suppresses breast cancer clonogenicity. **a**, **b** After transfecting MCF-7 cells with the circTADA2A-E6 vector, siRNA #1 or #2, miR-203a-3p mimic, or miR-203a-3p inhibitor, colony formation was analyzed after 14 days (left, representative pictures; right, quantitative bar for colony numbers). Error bars represent mean ± SEM from three independent experiments. *p < 0.05, **p < 0.01. Inh indicates inhibitor. **c** Schematic representation of the balance of circTADA2A-E6/miR-203a-3p/*SOCS3* axis in normal mammary gland cells and its imbalance in breast cancer cells. In normal cells, miR-203a-3p binds to either circTADA2A-E6 or its target gene *SOCS3* to ensure certain amount expression of *SOCS3* to maintain the homeostasis. Once circTADA2A-E6 is significantly downregulated, majority amount of miR-203a-3p would bind to *SOCS3* 3′UTR to post-transcriptionally repress the expression of *SOCS3*. This imbalance could have an important role in tumorigenesis

To explore the molecular mechanism of circTADA2A-E6 anti-tumor activity for breast cancer progression and metastasis, we first explored the localization of circTADA2A-E6. As shown in Fig. 5a, endogenous circTADA2A-E6 mainly resided in the cytoplasm in MDA-MB-231 cells, and the ectopic circTADA2A-E6 was consistently located with endogenous circTADA2A-E6. Next, we predicted the potential miRNAs binding to circTADA2A-E6 using Arraystar circRNA programs, CircNet and CircInteractome. We observed that circTADA2A-E6 has multiple potential binding sites for the miR-214 family, the miR-302 family, miR-203a-3p, the miR-342 family, and miR-197–5p (Fig. 5b). We confirmed binding interactions between miR-203a-3p and miR-302c-3p with circTADA2A-E6 by demonstrating a reduction in luciferase activity by at least 10 and 15% for miR-203a-3p and miR-302c-3p, respectively, and 25% for miR-203a-3p and miR-302c-3p in combination (Fig. 5c). Also, fluorescence in situ hybridization (FISH) analysis confirmed the co-localization of circTADA2A-E6 and miR-203a-3p in the cytoplasm of MCF-7 (Fig. 5d). Next, 81 mRNAs (60 miR-203a-3p-targeting mRNAs and 21 miR-302c-3p-targeting mRNAs) were predicted by TargetScan and PITA; the regulatory networks of circTADA2A-E6/miR-203a-3p and miR-302c-3p/mRNA axes are shown in Fig. 5e. The *SOCS3* gene was predicted as a downstream target gene of the circTADA2A-E6/miR-203a-3p axis. Interestingly, an early study reported that miR-203a-3p promotes cell proliferation by targeting *SOCS3* in MCF-7 cells^35^. As shown in Fig. 5f, we demonstrated that the expression of *SOCS3* was regulated by circTADA2A-E6 sponging of miR-203a-3p. *SOCS3* expression was significantly increased by ectopic circTADA2A-E6 expression (lanes 4–5) and miR-203a-3p inhibition (lanes 6–7). In contrast, *SOCS3* expression was suppressed by circTADA2A-E6 siRNAs (lanes 1–3) or miR-203a-3p mimic (lanes 7–8) and circTADA2A-E6-induced *SOCS3* expression was prevented by adding a miR-203a-3p mimic (lanes 9–11). Moreover, circTADA2A-E6-mediated suppression of colony formation was rescued by adding miR-203a-3p mimic or *SOCS3* siRNA (Fig. 6a). Conversely, circTADA2A-E6 siRNA-mediated colony formation was attenuated by adding miR-203a-3p mimics (Fig. 6b). Intriguingly, the strong correlation was found between the ability of colony formation and the expression of circTADA2A-E6 and miR-203a-3p: when circTADA2A-E6 or circTADA2A-E6 + miR-203a-3p mimic group showed its ability for the inhibition of colony formation, the expression of circTADA2A-E6 was increased about 90.53- or 85.51-fold, respectively (Supplementary Figure S3a); the expression of miR-203a-3p was increased about 124.32-fold in circTADA2A-E6 + miR-203a-3p mimic group (Supplementary Figure S3b). In addition, we performed qRT-PCR to study the effect of miR-203a-3p on circTADA2A-E6, and the result showed that miR-203a-3p had no influence on the regulation of circTADA2A-E6 (Supplementary Figure S3c and S3d, *p* < 0.05). Collectively, these data supported the idea that circTADA2A-E6 functions as miRNA sponge to protect *SOCS3* from miR-203a-3p-mediated suppression.

To address the potential clinical implications of the miR-203a-3p and its target gene *SOCS3* in breast cancer, we analyzed the relationship between miR-203a-3p and its target gene *SOCS3* and their correlation with RFS/OS of patients (Kaplan–Meier plotter, http://kmplot.com, *N* = 5143). As shown in Supplementary Figure S4, patients with higher levels of miR-203a-3p had poor prognosis as evidenced by OS in all patients (*p* = 0.026, HR = 1.44) (Figure S4a) or in TNBC (*p* = 0.008, HR = 4.73) (Figure S4b), and patients with lower levels of *SOCS3* had poor prognosis as evidenced by OS in all patients (*p* = 0.0036, HR = 0.63) (Figure S4c) or in TNBC (*p* = 0.00067, HR = 0.33) (Figure S4d), and RFS in all patients (*p* = 5.1e−08, HR = 0.65) (Figure S4e) or in TNBC (*p* = 0.0025, HR = 0.61) (Figure S4f). These results were consistent with the recent report that reduced expression of *SOCS3* is closely related to lymph node metastasis^36^. Higher mRNA expression levels of *SOCS3* are significantly associated with earlier tumor stage and better clinical outcome in human breast cancer^37^.

## Discussion

Understanding the crucial roles and functions of circRNAs is becoming a novel focus in cancer research. To date, a few circRNAs have been identified as biomarkers for cancer^23,24,26,32^. RNA sequencing and microarray analyses have been widely used to define the patterns of expressed circRNAs in various cancers. Galasso et al.^33^ first discovered a panel of predicted circRNAs by analyzing RNA sequencing data from five paired breast cancer samples. Lu et al.^24^ identified 1155 differentially expressed circRNAs from microarray analysis of four matched invasive ductal breast cancer and normal breast tissues. RNA sequencing data in The Cancer Genome Atlas (TCGA) from a cohort of breast cancer (*n* = 885) and adjacent untransformed tissue samples (*N* = 13) were analyzed by Nair et al.^34^, who reported 256, 288, and 411 tumor-specific circRNAs for TNBC, ER-positive, and Her-2- positive breast cancer subtypes, respectively. Furthermore, several studies reported that circRNAs are associated with cell cycle in breast cancer^17,38^ and with tight junctions, antigen presentation, and mTOR signaling pathways in TNBC^34^. Thus, circRNAs may be significantly involved in breast cancer biological processes.

In the present study, by comparing circRNA microarray data obtained from four TNBC and LA breast cancer samples each with three normal mammary gland tissues, we identified 215 and 73 differentially expressed circRNAs, respectively, out of which 140 were upregulated and 95 downregulated. 465 miRNAs demonstrated potential binding to these differentially expressed circRNAs. KEGG and GO analysis of the circRNAs/miRNAs/mRNA axis demonstrated that these networks are potentially involved in several biological processes in breast cancer: for example, (i) circCDC27 could be involved in ErbB signaling and apoptosis pathways (Supplementary Figure S5a) and (ii) circTADA2A-E6 could be involved in the PI3K-AKT, mTOR signaling pathway, ECM–receptor interaction, and apoptosis (Supplementary Figure S5b).

We focused on addressing the roles of two circTADA2As that were consistently and significantly decreased in breast cancer, which were reported differentially expressed in colon cancer and hepatocellular carcinoma^25,34,39^. In the present study, we confirmed decreased expression of two circTADA2As in 178 breast cancer patients’ specimens as well as 10 breast cancer cell lines. More importantly, we further defined clinical relevance of circTADA2A expression in 115 TNBCs. As compared to normal subjects, ~84.7–94.5% of breast cancer patients had lower expression levels of both circTADA2A-E6 and circTADA2A-E5/E6 (Fig. 2e, f and Supplementary Figure S6a, b). Thus, these data indicate that circRNAs might have potential diagnostic value for breast cancer. This is consistent with a recent report that circRNAs have high diagnostic value for breast cancer (AUC = 0.82, combined hsa_circ_006054, hsa_circ_100219 and hsa_circ_406697)^24^.

circRNAs have been demonstrated to have significant effects on cancer cell proliferation^25,26,40,41^, migration and invasion^25,26,40^, anchorage-independent growth^25^, and cell cycle progression^40,41^. Reduced levels of circRNAs in the ER-positive subtype were negatively correlated with the risk-of-relapse proliferation (ROR-P) score for proliferating genes^34^. Our results are in agreement with those of Yang et al.^42^ showing higher expression levels of individual circRNAs, i.e., circTADA2As and circFOXO3, in the non-cancerous cell line MCF-10A compared with breast cancer cells. circTADA2A-E6 expression knockdown promoted breast cancer cell proliferation, colony formation, and migration and invasion. Collectively, these findings supported the hypothesis that circRNAs have important roles in breast cancer progression.

Increasing evidence points to an association between circRNA expression and clinicopathological parameters of various cancers, such as tumor size in HCC^43^, cell differentiation in gastric cancer^32^, and lymph node metastasis and tumor staging in NSCLC^44^. In TNBC patients, ours is the first report to show association of decreased circTADA2A-E6 with lymph node metastasis and advanced clinical stage. Furthermore, previous findings suggested that circRNAs serve as prognostic biomarkers for malignancies, such as circMTO1 and circCCDC66 for HCC^25,26^ and circPVT1 for gastric cancer^45^. Consistent with these reports, our present study demonstrated that TNBC patients with low level of circTADA2A-E6 had shorter OS and DFS. Most importantly, after univariate and multivariate analyses, circTADA2A-E6 stood out as a potential independent prognostic marker for both DFS and OS.

Prior studies have established that circRNAs can act as sponges to miRNAs^19,46^ to promote cancer cell proliferation and invasion^20,26,45^. Herein, we identified the role of circTADA2A-E6 as a sponge for miR-203a-3p and miR-302c-3p. Significantly, miR-203a-3p was upregulated in 10 paired breast cancer/normal tissues^47^, promoted colony formation, transformation and migration of breast cancer cells^47^, and was a predictor of poor prognosis for breast cancer patients^48,49^. Furthermore, *SOCS3*, a target gene of miR-203a-3p^50^, was downregulated in breast cancer tissues and was a good predictor for lymph node metastasis in breast cancer^36^. Overexpression of *SOCS3* had an anti-proliferative effect on breast cancer cells^51^. In our study, *SOCS3* was predicted as a downstream target gene of the circTADA2A-E6/miR-203a-3p network. We demonstrated that circTADA2A-E6-induced *SOCS3* expression was prevented by miR-203a-3p mimics and circTADA2A-E6-induced suppression of colony formation was rescued by addition of a miR-203a-3p mimic. These results strongly support the contention that the circTADA2A-E6/miR-203a-3p/*SOCS3* axis has an important role in the inhibition of breast cancer progression (Fig. 6c). For further translation exploration for targeting circTADA2A-E6 axis in cancer therapeutic, there are some issues need to be firstly taken into consideration, such as which gene is suitable as a druggable target. We showed that long-term expression of circTADA2A-E6 in breast cancer cells and 293T resulted in strong cellular toxic. Thus, stable circTADA2A-E6 expressing cell lines could not be established or obtained (data not shown) in cell lines we used. Based on recent studies, novel findings that elevated level of circTADA2A-E6 might be detrimental to non-cancerous cells or normal mammary gland cells, too. In addition, miR-203a-3p could be considered as an alternative target. To complete all the roles of circTADA2A-E6 during tumorigenesis, we might find more potential druggable targets for breast cancer.

In summary, by using circRNA screening and functional verification together with clinical evidence, our study demonstrated the potential of two circTADA2As as promising prognostic biomarkers for breast cancer. Especially, circTADA2A-E6 functioned as a tumor suppressor by inhibiting cell proliferation, migration, and metastasis in breast cancer. Mechanistically, circTADA2A-E6 acted as a miR-203a-3p sponge restoring the expression of the miR-203a-3p target gene *SOCS3*. To the best of our knowledge, this is the first report thoroughly investigating the biological functions of circTADA2As and their clinical implications in breast cancer. This study may have a fundamental influence on breast cancer research, particularly for the identification of new biomarkers and future therapeutic targets paving the way for new pharmaceutical interventions for breast cancer.

## Materials and methods

### Patient enrollment

A total of 121 breast cancer tissues (LA, *N* = 25; LB, *N* = 21; Her-2, *N* = 17; TNBC, *N* = 58) and 16 normal mammary gland tissues were collected from breast cancer surgical procedures between June 2009 and December 2015 at Shantou University Medical College affiliated with Cancer Hospital, Shantou, China. An additional 57 TNBC samples were collected from breast cancer surgical specimens archived between 2006 and 2011 at Zhejiang cancer hospital, Hangzhou, China and Linyi People’s Hospital, Linyi, China. All fresh tumor tissue specimens were immediately preserved in RNAlater^®^ RNA Stabilization Solution (Invitrogen, Carlsbad, CA, USA), and stored long-term at −80 °C. Stage and classification of collected tumors and normal mammary gland tissues were histologically verified by pathologists (Supplementary Figure S1). The study was approved by the ethics committee of the Cancer Hospital of Shantou University Medical College, Zhejiang Cancer Hospital and Linyi People’s Hospital, and written informed consent was obtained from all patients involved in this study, and clinical follow-up was performed through November 2016.

### circRNA microarray hybridization

Sample preparation and microarray hybridization were performed based on the Arraystar’s standard protocols (Arraystar, Inc., Rockville, MD, USA). Briefly, total RNA was digested with RNase R (Epicentre, Inc., Madison, WI, USA) to remove linear RNAs and enrich circRNAs. Subsequently, the enriched circRNAs were amplified and transcribed into fluorescent labeled cRNAs using a random priming method. The labeled cRNAs were purified with an RNeasy Mini Kit (Qiagen, Hilden, Germany). The concentrations and specific activities of labeled cRNAs were measured by NanoDrop ND-1000 (Thermo Scientific, Waltham, MA, USA). Labeled cRNAs were hybridized onto the Arraystar Human circRNA Chip (8 × 15 K; Arraystar, Inc., Rockville, MD, USA) containing 5396 probes for human circRNAs. Arrays were scanned using an Agilent Scanner G2505C (Agilent, Santa Clara, California). Data were extracted using Agilent Feature Extraction Software. A series of data processing, including quantile normalization, was performed using the R software package. Differentially expressed circRNAs were selected according to the fold-change cutoff (FC ≥ 1.5) and *p* value with statistical significance (*p* ≤ 0.05). Microarray-seq data have been deposited at Gene Expression Omnibus (GSE101124).

### Bioinformatic analysis of circRNA/miRNA/mRNA network

The association for circRNA/miRNA was predicted by Arraystar circRNA program, CircNet, and CircInteractome (Supplementary Table 5). Subsequently, TargetScan and PITA were used to find miRNAs and their target genes. Accordingly, Cytoscape was applied to build circRNA/miRNA/mRNA networks. KEGG and GO analysis of circRNAs/miRNA/mRNA axis was described in Supplementary Materials and Methods.

### Cell lines, oligos, and plasmids

The information of cell lines was shown in Supplementary Materials and Methods. Specific knockdown of circTADA2A-E6 was achieved using three siRNA oligonucleotides designed and synthesized by Ribobio, Guangzhou, China to target the back-splice junction (Supplementary Table 6). To efficiently circularize a circRNA transcript in cells, the mature sequence for circTADA2A-E6 (chr17, 35800605–35800763) was synthesized and cloned into pLCDH-CMV-MCS-EF1-copGFP-puro (Geneseed; Guangzhou, China, Supplementary Figure S7a). A luciferase reporter incorporating the circTADA2A-E6 sequence (phRluc-circTADA2A-E6; Supplementary Figure S7b) was constructed by subcloning the circTADA2A-E6 fragment into the 3′-UTR downstream region of *Renilla* luciferase in the pciCHECK^TM^-2 vector (Geneseed; Guangzhou, China). Primer sequences for phRluc-circTADA2A-E6 subcloning are shown in Supplementary Table 6.

### Transient transfection and functional assay

For transient transfections, Lipofectamine 3000 (Invitrogen, Carlsbad, CA) and Opti-MEM (Gibco, Carlsbad, CA) were used according to the manufacturer’s instructions. For transfection studies, 5 μg per 60 mm dish of circTADA2A-E6 expression vector, 100 nM circTADA2A-E6 siRNA or 100 nM miR-203a-3p mimic or inhibitor (Supplementary Table 6) was used for the functional assays. The details of cell proliferation assay, colony formation assay, cell invasion, migration, and wound healing assay are shown in Supplementary Materials and Methods.

### Xenograft animal studies

All animal protocols were approved by the Animal Care and Use Committee of Shantou University Medical College (SUMC). Five-week-old female NU/NU Nude mice were purchased from Beijing Vital River Laboratory Animal Technology (Vital River, Beijing, China). The details are shown in Supplementary Materials and Methods.

### Quantitative reverse transcription polymerase reaction, western blotting, and luciferase reporter assay

Total RNA was extracted using Trizol reagent (Life Technologies, Carlsbad, CA, USA). Purity and concentration of RNA samples were determined with the NanoDrop ND-1000. RNA integrity was assessed by electrophoresis on a denaturing agarose gel. cDNAs were synthesized from total RNA using Geneseed® II First Strand cDNA Synthesis Kit (Geneseed, Guangzhou, China). The sequences of eight selected circRNAs were acquired from the CircInteractome database. Primers used in qRT-PCR were designed as convergent primers to detect circular junctions (Supplementary Table 6 and Supplementary Figure S8). The expected PCR products for individual circRNAs were determined via melting curve analysis (Supplementary Figure S9), and the splicing junction was confirmed by sequencing (Supplementary Figure S10). β-Actin served as an internal control. The 2^−ΔΔCt^ method was used to quantitate gene expression. In addition, the details for WB and luciferase reporter assay are shown in Supplementary Materials and Methods.

### Fluorescence in situ hybridization

The cell suspension was pipetted onto autoclaved glass slides. After prehybridization (1×PBS/0.5% Triton X-100), cells were hybridized in hybridization buffer (4% formamide, 10% Dextran sulfate, 1×Denhardt’s solution, 2×SSC, 10 mM DDT, 1 mg/ml yeast transfer RNA, 1 mg/ml sheared salmon sperm DNA) with specific probes at 60 ℃ overnight. Cy3-labeled probes specific to circTADA2A-E6 and FITC-labeled probes specific to miR-203a-3p (Geneseed; Guangzhou, China, Supplementary Table 6) were used in the hybridization. Nuclei were counterstained with 4,6-diamidino-2-phenylindole. The images were acquired on a Laser Scanning Confocal Microscope (Leica TCS SP2) (Leica Microsystems, Mannheim, Germany).

### Statistical analysis

Statistical analyses were performed using SPSS software (IBM, Armonk, NY, USA). Statistically significant differences were calculated using Student’s *t*-tests and one-way analysis of variance (ANOVA) for parametric and nonparametric data, respectively. Receiver operating characteristic (ROC) analysis was applied to evaluate the power of circTADA2A-E6 expression as a breast cancer diagnostic marker, and the AUC was calculated in Prism 5.0 (Microsoft, Redmond, WA, USA). Survival curves for DFS and OS were generated using the Kaplan–Meier method. To investigate the clinicopathologic factors related to recurrence and metastasis, Cox regression analyses (univariate and multivariate) were performed. *p* *<* 0.05 was considered statistically significant for all statistical analyses.

## Supplementary information

Supplemental figure legends

Supplementary file 1 Materials and Methods

Supplementary file 2 Supplementary Tables

Supplementary file 3 Supplementary Figures
