# Supplementary material for: circTADA2As suppress breast cancer progression and metastasis via targeting miR-203a-3p/SOCS3 axis
Source: Cell Death Dis. 2019 Feb 20;10(3):175. doi: 10.1038/s41419-019-1382-y (PMC6382814; doi:10.1038/s41419-019-1382-y)
Supplement: Supplementary file 1 — Supplemental figure legends [file 41419_2019_1382_MOESM1_ESM.docx]

**Supplementary Figure lengends**

**Figure S1.** H&E staining of human normal mammary gland tissues (N = 11).

**Figure S2.** Images of isolated tumors.

**Figure S3.** The relative expression of circTADA2A-E6 and miR-203a-3p.

**Figure S4.** Kaplan–Meier survival curve analysis for the correlation between has-miR-203a-3p or *SOCS3* expression and overall survival (OS) in all patients (a, c) or in TNBC (b, d). The correlation between *SOCS3* expression and recurrence-free survival (RFS) in all patients (e) and in TNBC (f).

**Figure S5.** KEGG and GO analysis of circRNAs/miRNA/mRNA axis profiles. (a) The blinking showing the target genes could be regulated by circCDC27/miR-7 or miR-1276/mRNA axis. (b) Signaling pathway enriched by circTADA2A-E6/miR-203a-3p and miR-302c-3p/mRNA axis.

**Figure S6.** ROC analysis for circTADA2A-E6 (a) and circTADA2A-E5/E6 (b) in LA, LB, and Her-2 breast cancer subtypes.

**Figure S7.** Schematic representation for circTADA2A-E6 vectors. (a) pLCDH-CMV-circTADA2A-EF1-copGFP-puro. (b) phRluc-circTADA2A-E6.

**Figure S8.** Schematic representation of the eight circRNAs formed by gene exon splicing. The arrows stand for divergent primers locus for qRT-PCR.

**Figure S9.** Melting curve of qRT-PCR showing a single, distinct product of the expected size for eight individual circRNAs.

**Figure S10.** Sanger sequencing showing head-to-tail splicing for eight individual circRNAs. Sanger sequencing for qRT-PCR product confirmed the existing of a head-to-tail junction, the red lines indicated the backsplice junction.
