# Supplementary material for: circTADA2As suppress breast cancer progression and metastasis via targeting miR-203a-3p/SOCS3 axis
Source: Cell Death Dis. 2019 Feb 20;10(3):175. doi: 10.1038/s41419-019-1382-y (PMC6382814; doi:10.1038/s41419-019-1382-y)
Supplement: Supplementary file 2 — Supplementary file 1 Materials and Methods [file 41419_2019_1382_MOESM2_ESM.pdf]

Supplementary Materials and Methods

Cell culture

Cells used in this study (MCF-7, MDA-MB-231, T47D, BT20, BT549, SKBR3, MDA-MB-157, MDA-MB-435, MDA-MB-436, MDA-MB-468, MCF-10A, 293T) were purchased from ATCC and cultured under the conditions according to ATCC recommendation, in a humidified atmosphere with 5% CO_2 at 37ºC. In some experiments, MCF-7 or MDA-MB-231 cells were serum-starved for 24 h （DMEM supplemented with 0 % bovine albumin）. MCF-10A were cultured in DMEM (Gibco-BRL, Carlsbad, CA, USA) supplemented with 5 % horse serum (Invitrogen), 0.5 % hydrocortisone (Sigma-Aldrich, St. Louis, MO, USA), 100 ng/ml cholera toxin (Sigma-Aldrich), 10 μg/ml insulin (Sigma-Aldrich), and 20 ng/ml recombinant human epidermal growth factor (Sigma-Aldrich). 293T cells were maintained in DMEM medium supplemented with 10% FBS (Gibco-BRL, Carlsbad, CA, USA).

Western blotting analysis

Total proteins were extracted 72 h after transfection. The cells in each well were lysed in 100 μl of lysis buffer containing RIPA (99 μl) and protease inhibitor cocktail (1 μl). Then, 60 micrograms of protein were resolved by SDS–PAGE analysis and transferred onto a PVDF membrane. The primary antibodies used were anti-E-Cadherin Ab (1:3000, Cell Signaling Technology, US), anti-Vimentin Ab (1:2000, Cell Signaling Technology, US), anti-SOCS3 Ab (1:1000, Cell Signaling Technology, US), anti-β-actin Ab（1:3000, ZSGB-Bio, China）. The membranes were washed and then incubated with Peroxidase-Conjugated Goat anti-Rabbit IgG (H+L)

(1: 3000; ZSGB-BIO) or Peroxidase-Conjugated Goat anti- Mouse IgG (H+L) (1: 3000; ZSGB-BIO) for 2 h at room temperature. The blots were visualized by ECL detection (Thermos Scientific).

MiRNA quantitative PCR

Total RNA was extracted using Trizol reagent as previous statement. Total RNA was reverse-transcribed into first-strand cDNA using a specific reverse transcription primer (miR-203a-3p: GTCGTATCCAGTGCAGGGTCCGAGGT ATTCGCACTGG ATACGACCCTAGTGGT; U6: GTCGTATCCAGTGCAGG GTCCGAGGTATTCG CACTGGATACGACCAAATATGGAAC) using Geneseed® II First Strand cDNA Synthesis Kit (Geneseed, Guangzhou, China), and cDNA was used as a template for the PCR reaction with a forward primer specific to the mature miR-203a-3p sequence (Supplementary Table 6). U6 served as an internal control.

***In vitro* cellular function assays**

CCK-8 assay for cell proliferation. The proliferation of MCF-7 and MDA-MB-231 cells was tested by CCK-8 kit (Beyotime, China). Approximately 48 hours later ，transfected 2 x 10^3 cells per well were seeded in triplicate in 96-well plates. At 24, 48, 72 and 96 hours, the CCK-8 reagent was added to each well and incubated at 37℃ for 1.5 h. The optical density at 450 nm was measured using an automatic microplate reader (Synergy4; BioTek, Winooski, VT, USA).

Transwell assay for cell invasion. Cell culture inserts (8 um pore size; BD) and matrigel invasion chamber (BD) were used to perform invasion assay, respectively. Transfected cells were serum-starved for 24 hours and 1×10^4 MDA-MB-231

transfected cells or 2×10^4 MCF-7 transfected cells in serum-free medium were seeded in the upper chamber. Cells were fixed with 4% paraformaldehyde for invasion assays at 24 hours for MDA-MB-231 or 48 hours for MCF-7, respectively., then cells were stained with 0.1% crystal violet. Each assay was performed in triplicate. The number of cells from 3 fields in each well was counted by two investigators.

Wound healing assay for cell migration. The injury line was made with a tip 2 mm wide on cells plated in culture dishes at 100% confluency. After rinsing with phosphate-buffered saline, transfected cells were allowed to migrate in complete medium and photographs were taken after 24 hours for MDA-MB-231 or 48 hours for MCF-7. An average of five random width of injury line was measured for quantitation.

Colony formation assay. For the colony formation assay, 400 transfected cells of MDA-MB-231 or 400 transfected cells of MCF-7 per well were plated in a 6-well plate in triplicate, and allowed to grow until visible colonies appeared. Colonies were fixed with 4% paraformaldehyde and stained with 0.1% crystal violet and then counted.

Rescue experiment. For rescue experiment, the siRNAs or plasmids of circTADA2A-E6 were transfected into MCF-7 cells, and after 12 hours, miR-203a-3p inhibitors or mimics were transfected into above-mentioned cells, and then 48 hours later, cells were seeded into 6-well plates for colony formation assay, or 72 hours later, cells were lysed for protein lysates.

Xenograft animal studies

Five-week-old female NU/NU Nude mice were purchased from Beijing Vital River Laboratory Animal Technology and kept under controlled conditions (12 h light and dark cycles). The animal protocol was approved by the institutional animal care and use committee of Medical College of Shantou University. MDA-MB-231 cells (1×10^7 cells in a volume of 100 μL PBS solution) were inoculated subcutaneously into the right fat pad and all the animals were acclimatised to experimental condition for a week before the injections. The treatment was started when the tumor volumes were about 100 mm^3 and the mice were randomly assigned into three groups and received intratumoral injection of siRNA NC, circTADA2A-E6 siRNA #1, #2 mixed with Entranster^{TM} -in vivo (Engreen Biosystem Co, Ltd, China). The dose of siRNA was 0.5 μg/g weight and the preparation of mixtures were performed following the manufacturer's protocols. The treatment injections for mice in each group were administrated every 7 days for two times and the tumor volume ($V=L\times W^2/2$, where L represents the longest diameter and W represents the shortest diameter) were recorded each time before the injection. All the animals were sacrificed at the end of the experiments.

Luciferase reporter assay

Cells were seeded in 12-well plates in triplicate at a density of 1×10^5 cells per well 24 h before transfection. The cells were co-transfected with a mixture of 1ug phRluc-circTADA2A-E6, and miRNA mimics at the indicated concentration. After 48 h, the luciferase activity was measured with a dual luciferase reporter assay system (Promega, Madison, WI). For comparison, Renilla measurements were normalized to

Firefly measurements. The effect of each miRNA on luciferase reporter with circTADA2A-E6 3'-UTR was then normalized with the effect of miRNA NC on luciferase reporter with circTADA2A-E6 3'-UTR. The fold change was calculated by each miRNA compared with NC. Each transfection experiment was repeated at least 3 times.

KEGG and GO analysis of circRNAs/miRNA/mRNA axis profile.

The predicted gene functions in the networks were annotated using GO and KEGG pathway analysis. Biological pathways were identified by Database for Annotation, Visualization and Integrated Discovery (DAVID: http://www.david.abcc.ncifcrf.gov/).
