# Supplementary material for: circTADA2As suppress breast cancer progression and metastasis via targeting miR-203a-3p/SOCS3 axis
Source: Cell Death Dis. 2019 Feb 20;10(3):175. doi: 10.1038/s41419-019-1382-y (PMC6382814; doi:10.1038/s41419-019-1382-y)
Supplement: Supplementary file 3 — Supplementary file 2 Supplementary Tables [file 41419_2019_1382_MOESM3_ESM.pdf]

Table S1 Patient and healthy adult information for the circRNA microarray study

No.	Molecular status	Age	Ethic	Clinical Stage
1	Health adult	42	Chinese Han	
2	Health adult	47	Chinese Han	
3	Health adult	37	Chinese Han	
4	LA	42	Chinese Han	$pT_2N_1M_0$ Ⅱb
5	LA	43	Chinese Han	$pT_2N_0M_0$ Ⅱa
6	LA	72	Chinese Han	$pT_2N_1M_0$ Ⅱb
7	LA	55	Chinese Han	$pT_2N_0M_0$ Ⅱa
8	TNBC	62	Chinese Han	$pT_2N_1M_0$ Ⅱb
9	TNBC	50	Chinese Han	$pT_1N_0M_0$ Ⅰa
10	TNBC	38	Chinese Han	$pT_2N_0M_0$ Ⅱa
11	TNBC	52	Chinese Han	$pT_1N_0M_0$ Ⅰa

Table S2. Differentially-expressed circRNAs in TNBC as compared to normal mammary gland tissues

Probe ID	circRNA_type	Chromosome	FC (abs)	Regulation	Gene Symbol
ASCRP000048	intronic	chr6	2.297	up	BACH2
ASCRP000086	antisense	chr18	2.383	up	DLGAP1
ASCRP000137	intragenic	chr15	2.160	up	EDC3
ASCRP000156	intragenic	chr3	1.592	up	TRA2B
ASCRP000232	antisense	chr12	1.617	up	EP400NL
ASCRP000259	antisense	chr14	3.124	up	RPPH1
ASCRP000262	antisense	chr15	1.806	up	TLE3
ASCRP000269	intronic	chr22	1.865	up	DGCR8
ASCRP000315	intragenic	chr14	5.790	up	RPPH1
ASCRP000334	intronic	chr6	1.912	up	ARID1B
ASCRP000343	intragenic	chr14	11.225	up	RPPH1
ASCRP000354	intronic	chr11	2.115	up	OSBPL5
ASCRP000360	intragenic	chr9	1.584	up	SNORA43
ASCRP000390	intragenic	chr14	3.252	up	RPPH1
ASCRP000421	exonic	chr1	1.715	up	RERE
ASCRP000574	exonic	chr1	1.517	up	SCMH1
ASCRP000601	exonic	chr1	2.097	up	STIL
ASCRP000657	exonic	chr1	2.383	up	LPHN2
ASCRP000760	exonic	chr1	2.732	up	UCK2
ASCRP000791	exonic	chr1	1.584	up	ABL2
ASCRP000808	exonic	chr1	1.876	up	IPO9
ASCRP000823	exonic	chr1	2.219	up	KCNH1
ASCRP000837	exonic	chr1	2.078	up	PTPN14
ASCRP000910	exonic	chr10	1.803	up	WDR37
ASCRP000958	exonic	chr10	3.615	up	WAC
ASCRP000961	exonic	chr10	1.733	up	ARHGAP12
ASCRP001003	exonic	chr10	1.928	up	KAT6B
ASCRP001004	exonic	chr10	1.723	up	KAT6B
ASCRP001052	exonic	chr10	1.793	up	PCGF6
ASCRP001174	exonic	chr11	1.631	up	TTC17
ASCRP001179	exonic	chr11	1.784	up	AMBRA1
ASCRP001226	exonic	chr11	1.817	up	DPP3
ASCRP001229	exonic	chr11	1.603	up	C11orf80
ASCRP001234	exonic	chr11	1.539	up	LRP5
ASCRP001248	exonic	chr11	1.917	up	NADSYN1
ASCRP001353	exonic	chr11	2.047	up	STT3A
ASCRP001380	exonic	chr12	2.508	up	A2ML1
ASCRP001429	exonic	chr12	2.151	up	LETMD1
ASCRP001523	exonic	chr12	1.867	up	SLC24A6
ASCRP001596	exonic	chr13	4.570	up	TNFRSF19
ASCRP001668	exonic	chr13	1.597	up	TMCO3
ASCRP001705	exonic	chr14	2.382	up	SEC23A
ASCRP001809	exonic	chr15	1.907	up	HERC2P3
ASCRP002054	exonic	chr16	1.927	up	RSL1D1
ASCRP002090	exonic	chr16	3.231	up	C16orf62
ASCRP002094	exonic	chr16	2.380	up	C16orf62
ASCRP002126	exonic	chr16	1.783	up	XPO6
ASCRP002171	exonic	chr16	2.069	up	CBFB
ASCRP002179	exonic	chr16	2.209	up	NFATC3
ASCRP002212	exonic	chr16	1.667	up	IST1
ASCRP002309	exonic	chr17	2.370	up	NEURL4
ASCRP002311	exonic	chr17	1.744	up	NEURL4
ASCRP002354	exonic	chr17	1.964	up	SSH2
ASCRP002369	exonic	chr17	1.893	up	RHOT1
ASCRP002394	exonic	chr17	1.528	up	MED1
ASCRP002404	exonic	chr17	1.602	up	NT5C3L
ASCRP002431	exonic	chr17	2.310	up	KIAA1267
ASCRP002438	exonic	chr17	1.622	up	NPEPPS
ASCRP002472	exonic	chr17	1.790	up	USP32
ASCRP002524	exonic	chr17	1.627	up	FBF1
ASCRP002539	exonic	chr17	2.160	up	PGS1
ASCRP002557	exonic	chr17	1.695	up	NPLOC4
ASCRP002561	exonic	chr17	1.570	up	ASPSCR1

ASCRP002611	exonic	chr18	1.761	up	PTPRM
ASCRP002644	exonic	chr18	2.118	up	C18orf8
ASCRP002652	exonic	chr18	2.981	up	ZNF521
ASCRP002734	exonic	chr19	2.099	up	MAP2K2
ASCRP002770	exonic	chr19	1.816	up	LDLR
ASCRP002800	exonic	chr19	2.608	up	PGLS
ASCRP002854	exonic	chr19	2.245	up	RYR1
ASCRP002876	exonic	chr19	1.636	up	GRIK5
ASCRP003019	exonic	chr2	1.904	up	THADA
ASCRP003066	exonic	chr2	1.604	up	GFPT1
ASCRP003071	exonic	chr2	1.981	up	CYP26B1
ASCRP003104	exonic	chr2	1.622	up	UXS1
ASCRP003147	exonic	chr2	1.723	up	TANK
ASCRP003197	exonic	chr2	1.506	up	SATB2
ASCRP003232	exonic	chr2	2.248	up	SERPINE2
ASCRP003255	exonic	chr2	2.499	up	INPP5D
ASCRP003261	exonic	chr2	1.670	up	UBE2F
ASCRP003426	exonic	chr21	1.591	up	DONSON
ASCRP003439	exonic	chr21	1.768	up	BACE2
ASCRP003472	exonic	chr22	1.517	up	MAPK1
ASCRP003562	exonic	chr22	1.629	up	TBC1D22A
ASCRP003620	exonic	chr3	2.129	up	CLASP2
ASCRP003650	exonic	chr3	1.542	up	SCAP
ASCRP003669	exonic	chr3	2.389	up	CDC25A
ASCRP003679	exonic	chr3	1.528	up	RBM5
ASCRP003746	exonic	chr3	1.876	up	PDIA5
ASCRP003753	exonic	chr3	1.502	up	ZNF148
ASCRP003849	exonic	chr3	2.006	up	XXYLT1
ASCRP003922	exonic	chr4	1.668	up	APBB2
ASCRP004044	exonic	chr4	3.231	up	LRBA
ASCRP004102	exonic	chr5	2.182	up	NUP155
ASCRP004124	exonic	chr5	1.523	up	PARP8
ASCRP004150	exonic	chr5	1.767	up	CCNB1
ASCRP004184	exonic	chr5	1.636	up	MSH3
ASCRP004196	exonic	chr5	1.620	up	MCTP1
ASCRP004228	exonic	chr5	2.796	up	SEC24A
ASCRP004277	exonic	chr5	1.820	up	EBF1
ASCRP004379	exonic	chr6	1.788	up	TRERF1
ASCRP004465	exonic	chr6	1.778	up	AHI1
ASCRP004480	exonic	chr6	1.934	up	STXBP5
ASCRP004502	exonic	chr6	2.498	up	IPCEF1
ASCRP004599	exonic	chr7	2.460	up	HIBADH
ASCRP004647	exonic	chr7	1.998	up	WBSCR17
ASCRP004753	exonic	chr7	2.550	up	CREB3L2
ASCRP005008	exonic	chr9	1.538	up	UBAP2
ASCRP005012	exonic	chr9	1.701	up	UBAP2
ASCRP005085	exonic	chr9	1.762	up	CDC14B
ASCRP005086	exonic	chr9	1.615	up	CDC14B
ASCRP005129	exonic	chr9	2.161	up	HSDL2
ASCRP005132	exonic	chr9	1.622	up	SLC31A1
ASCRP005179	exonic	chr9	1.748	up	SPTAN1
ASCRP005187	exonic	chr9	2.320	up	NUP214
ASCRP005240	exonic	chrX	2.604	up	CASK
ASCRP005274	exonic	chrX	1.998	up	SLC25A43
ASCRP005286	exonic	chrX	1.758	up	FLNA
ASCRP005304	intronic	chr1	1.870	up	RPS8
ASCRP005307	intronic	chr1	2.262	up	GADD45A
ASCRP005324	intronic	chr16	1.924	up	ANKS3
ASCRP005364	intronic	chr22	1.738	up	MCM5
ASCRP000064	intronic	chr12	3.654	down	PRB4
ASCRP000075	antisense	chr15	1.556	down	KLF13
ASCRP000085	intronic	chr17	2.116	down	KIF18B
ASCRP000161	intronic	chr5	1.847	down	C5orf56
ASCRP000199	antisense	chr19	2.437	down	RPS15
ASCRP000212	antisense	chr4	5.275	down	MARCH1
ASCRP000266	intragenic	chr19	2.451	down	C19orf18
ASCRP000493	exonic	chr1	1.728	down	CEP85
ASCRP000594	exonic	chr1	1.789	down	GPBP1L1

ASCRP000661	exonic	chr1	2.122	down	RPAP2
ASCRP000741	exonic	chr1	2.677	down	GON4L
ASCRP000745	exonic	chr1	1.811	down	KIAA0907
ASCRP000775	exonic	chr1	1.530	down	PRRC2C
ASCRP000818	exonic	chr1	2.436	down	DSTYK
ASCRP000906	exonic	chr10	2.033	down	WDR37
ASCRP000987	exonic	chr10	2.218	down	DDX21
ASCRP001008	exonic	chr10	1.558	down	DLG5
ASCRP001059	exonic	chr10	4.007	down	FAM160B1
ASCRP001087	exonic	chr10	1.639	down	ZRANB1
ASCRP001144	exonic	chr11	1.553	down	ANO5
ASCRP001219	exonic	chr11	1.516	down	MARK2
ASCRP001241	exonic	chr11	2.664	down	CPT1A
ASCRP001277	exonic	chr11	1.800	down	ALG8
ASCRP001334	exonic	chr11	2.013	down	ARCN1
ASCRP001375	exonic	chr12	2.525	down	IFFO1
ASCRP001384	exonic	chr12	1.952	down	LRP6
ASCRP001410	exonic	chr12	2.593	down	DENND5B
ASCRP001430	exonic	chr12	2.654	down	CSRNP2
ASCRP001446	exonic	chr12	1.614	down	CS
ASCRP001538	exonic	chr12	2.563	down	RNF10
ASCRP001569	exonic	chr12	1.755	down	AACS
ASCRP001620	exonic	chr13	2.302	down	KIAA0564
ASCRP001667	exonic	chr13	2.653	down	TMCO3
ASCRP001740	exonic	chr14	4.279	down	PCNX
ASCRP001893	exonic	chr15	2.748	down	ANXA2
ASCRP002064	exonic	chr16	1.859	down	ABCC1
ASCRP002069	exonic	chr16	1.737	down	ABCC1
ASCRP002384	exonic	chr17	29.629	down	TADA2A
ASCRP002386	exonic	chr17	19.698	down	TADA2A
ASCRP002434	exonic	chr17	1.953	down	CDC27
ASCRP002523	exonic	chr17	1.561	down	UNK
ASCRP002556	exonic	chr17	1.845	down	NPLOC4
ASCRP002757	exonic	chr19	1.725	down	DNMT1
ASCRP002758	exonic	chr19	1.922	down	DNMT1
ASCRP002932	exonic	chr2	23.180	down	NOL10
ASCRP002964	exonic	chr2	5.719	down	ZNF512
ASCRP003001	exonic	chr2	2.627	down	CRIM1
ASCRP003002	exonic	chr2	2.840	down	STRN
ASCRP003092	exonic	chr2	1.962	down	LOC285033
ASCRP003107	exonic	chr2	1.995	down	SEPT10
ASCRP003189	exonic	chr2	2.449	down	HECW2
ASCRP003236	exonic	chr2	1.825	down	RHBDD1
ASCRP003290	exonic	chr20	3.447	down	PTPRA
ASCRP003424	exonic	chr21	1.622	down	IFNGR2
ASCRP003468	exonic	chr22	1.541	down	UBE2L3
ASCRP003491	exonic	chr22	1.525	down	EWSR1
ASCRP003569	exonic	chr22	1.590	down	RABL2B
ASCRP003607	exonic	chr3	2.112	down	RBMS3
ASCRP003643	exonic	chr3	8.013	down	SETD2
ASCRP003686	exonic	chr3	1.578	down	ACY1
ASCRP003776	exonic	chr3	1.514	down	IL20RB
ASCRP003872	exonic	chr3	2.069	down	LRCH3
ASCRP003973	exonic	chr4	1.587	down	SEC31A
ASCRP003994	exonic	chr4	1.847	down	HERC5
ASCRP004073	exonic	chr5	2.938	down	NSUN2
ASCRP004108	exonic	chr5	1.741	down	NUP155
ASCRP004135	exonic	chr5	2.121	down	DEPDC1B
ASCRP004219	exonic	chr5	1.908	down	VDAC1
ASCRP004255	exonic	chr5	1.649	down	DIAPH1
ASCRP004315	exonic	chr6	1.512	down	DUSP22
ASCRP004360	exonic	chr6	2.588	down	LRRC16A
ASCRP004467	exonic	chr6	1.650	down	PDE7B
ASCRP004489	exonic	chr6	1.571	down	LATS1
ASCRP004503	exonic	chr6	2.204	down	IPCEF1
ASCRP004532	exonic	chr6	1.819	down	MAP3K4
ASCRP004534	exonic	chr6	1.575	down	MAP3K4
ASCRP004576	exonic	chr7	2.397	down	PHF14

ASCRP004595	exonic	chr7	1.716	down	MPP6
ASCRP004736	exonic	chr7	1.540	down	NRF1
ASCRP004813	exonic	chr8	1.596	down	VPS37A
ASCRP004906	exonic	chr8	4.349	down	PTDSS1
ASCRP004947	exonic	chr8	1.762	down	TRAPPC9
ASCRP005005	exonic	chr9	1.575	down	UBAP2
ASCRP005047	exonic	chr9	1.895	down	TLE4
ASCRP005049	exonic	chr9	2.703	down	TLE4
ASCRP005164	exonic	chr9	1.745	down	NEK6
ASCRP005171	exonic	chr9	2.145	down	FAM125B
ASCRP005302	intronic	chr1	1.934	down	SNHG12
ASCRP005355	intronic	chr20	1.583	down	NINL
ASCRP005370	intronic	chr3	1.886	down	NISCH
ASCRP005383	intronic	chr6	2.062	down	C6orf132
ASCRP005384	intronic	chr6	1.754	down	TJAP1
ASCRP005396	intronic	chrX	1.502	down	RPS4X

Table S3. Validation list of eight downregulated circRNAs in TNBC from microarray data

Gene Symbol of circRNA origination	Location	Chrome	Size (bp)	Ratio (folds, down)
TADA2A	exonic 5 plus 6	chr17	250	29.62
NOL10	exonic	chr12	562	23.17
TADA2A	exonic 6	Chr17	158	19.69
NSUN2	exonic	Chr5	178	2.94
CSRNP2	exonic	Chr12	260	2.65
FAM125B	exonic	Chr9	581	2.14
CDC27	exonic	Chr17	843	1.95
ABCC1	exonic	Chr16	567	1.85

Table S4. Correlations between clinicopathological factors and circTADA2A-E5/E6 expression ($2^{-\Delta\Delta Ct}$) in TNBC

Characteristics	No.of patients (%)	Mean ± SEM	*p* value
Age			
≥50	67 (58.3%)	0.94489 ± 0.23150	0.819
<50	48 (41.7%)	0.85775 ± 0.31123	
AJCC TNM stage#			
Ⅰ - Ⅱ	82 (74.5%)	0.94605 ± 0.24210	0.660
Ⅲ - Ⅳ	28 (25.5%)	0.75018 ± 0.26800	
T classification##			
T_1	25 (24.0%)	0.48693 ± 0.16316	0.551
T_2	70 (67.3%)	1.14613 ± 0.29297	
T_3	5 (4.8%)	0.57056 ± 0.0.22684	
T_4	4 (3.8%)	0.64002 ± 0.26530	
Lymphatic metastasis			
N_{0-1}	91 (79.1%)	0.95593 ± 0.22138	0.623
N_{2-3}	24 (20.9%)	0.72874 ± 0.31091	

AJCC, American Joint Committee on Cancer; #, 4.35% patient information missing; ##, 9.56% patient information missing ; *p < 0.05

Table S5. Differentially-expressed circRNAs and target miRNAs

circRNA ID	Target miRNAs					Gene Symbol
hsa_circ_0001625	hsa-miR-580-5p	hsa-miR-139-3p	hsa-miR-877-3p	hsa-miR-603	hsa-miR-541-5p	BACH2
hsa_circ_0000822	hsa-miR-144-3p	hsa-miR-545-3p	hsa-miR-92a-2-5p	hsa-miR-625-5p	hsa-miR-18a-3p	DLGAP1
hsa_circ_0000632	hsa-miR-765	hsa-miR-744-3p	hsa-miR-33b-3p	hsa-miR-519e-3p	hsa-miR-515-3p	EDC3
hsa_circ_0001373	hsa-miR-218-5p	hsa-miR-148b-5p	hsa-miR-647	hsa-miR-378a-5p	hsa-miR-639	TRA2B
hsa_circ_0000463	hsa-miR-627-5p	hsa-miR-550a-5p	hsa-miR-515-3p	hsa-miR-499a-3p	hsa-miR-550a-3-5p	EP400NL
hsa_circ_0000516	hsa-miR-18b-3p	hsa-miR-670-5p	hsa-miR-23a-5p	hsa-miR-125a-3p	hsa-miR-125b-5p	RPPH1
hsa_circ_0000622	hsa-miR-296-3p	hsa-miR-1301-3p	hsa-miR-485-5p	hsa-miR-221-5p	hsa-miR-383-5p	TLE3
hsa_circ_0001204	hsa-miR-612	hsa-miR-657	hsa-miR-362-3p	hsa-miR-377-3p	hsa-miR-136-5p	DGCR8
hsa_circ_0000517	hsa-miR-127-5p	hsa-miR-326	hsa-miR-330-5p	hsa-miR-597-3p	hsa-miR-204-3p	RPPH1
hsa_circ_0001659	hsa-miR-203a-5p	hsa-miR-619-5p	hsa-miR-504-3p	hsa-miR-597-3p	hsa-miR-134-5p	ARID1B
hsa_circ_0000520	hsa-miR-328-3p	hsa-miR-1296-5p	hsa-miR-146b-3p	hsa-miR-330-5p	hsa-miR-608	RPPH1
hsa_circ_0000273	hsa-miR-651-3p	hsa-miR-383-5p	hsa-miR-340-5p	hsa-miR-129-5p	hsa-miR-33b-3p	OSBPL5
hsa_circ_0001903	hsa-miR-876-3p	hsa-miR-668-3p	hsa-miR-302a-3p	hsa-miR-138-5p	hsa-miR-762	SNORA43
hsa_circ_0000519	hsa-miR-328-3p	hsa-miR-1296-5p	hsa-miR-146b-3p	hsa-miR-330-5p	hsa-miR-671-5p	RPPH1
hsa_circ_0009577	hsa-miR-105-5p	hsa-miR-16-5p	hsa-miR-424-5p	hsa-miR-107	hsa-miR-103a-3p	RERE
hsa_circ_0004628	hsa-miR-21-3p	hsa-miR-136-5p	hsa-miR-657	hsa-miR-593-5p	hsa-miR-22-5p	SCMH1
hsa_circ_0000069	hsa-miR-345-5p	hsa-miR-29b-1-5p	hsa-miR-34a-5p	hsa-miR-134-3p	hsa-miR-892a	STIL
hsa_circ_0013048	hsa-miR-630	hsa-miR-605-3p	hsa-let-7g-5p	hsa-let-7i-5p	hsa-let-7a-5p	LPHN2
hsa_circ_0006758	hsa-miR-324-3p	hsa-miR-29a-5p	hsa-miR-580-3p	hsa-miR-485-3p	hsa-miR-149-5p	UCK2
hsa_circ_0006980	hsa-miR-130b-3p	hsa-miR-33b-3p	hsa-miR-197-3p	hsa-miR-550a-5p	hsa-miR-597-3p	ABL2
hsa_circ_0015928	hsa-miR-558	hsa-miR-215-3p	hsa-miR-103a-2-5p	hsa-miR-185-3p	hsa-miR-639	IPO9
hsa_circ_0006123	hsa-miR-125a-5p	hsa-miR-485-5p	hsa-miR-15a-3p	hsa-miR-214-3p	hsa-miR-199a-5p	KCNH1
hsa_circ_0007015	hsa-miR-766-3p	hsa-miR-642a-3p	hsa-miR-519d-5p	hsa-miR-326	hsa-miR-96-5p	PTPN14
hsa_circ_0000206	hsa-miR-488-5p	hsa-miR-511-5p	hsa-miR-489-3p	hsa-miR-449c-5p	hsa-miR-892b	WDR37
hsa_circ_0007503	hsa-miR-593-3p	hsa-miR-606	hsa-miR-520f-3p	hsa-miR-299-3p	hsa-miR-29b-1-5p	WAC
hsa_circ_0000231	hsa-miR-135b-5p	hsa-miR-135a-5p	hsa-miR-518c-5p	hsa-miR-552-3p	hsa-miR-30c-1-3p	ARHGAP12
hsa_circ_0005164	hsa-miR-302b-5p	hsa-miR-22-5p	hsa-miR-758-5p	hsa-miR-494-5p	hsa-miR-204-3p	KAT6B
hsa_circ_0005235	hsa-miR-22-5p	hsa-miR-758-5p	hsa-miR-494-5p	hsa-miR-410-5p	hsa-miR-302b-5p	KAT6B
hsa_circ_0019803	hsa-miR-200c-5p	hsa-miR-377-5p	hsa-miR-208a-5p	hsa-miR-367-5p	hsa-miR-599	PCGF6
hsa_circ_0021773	hsa-let-7b-5p	hsa-let-7i-5p	hsa-miR-380-5p	hsa-miR-15b-3p	hsa-miR-182-5p	TTC17
hsa_circ_0007001	hsa-miR-494-5p	hsa-miR-139-3p	hsa-miR-765	hsa-miR-410-5p	hsa-miR-642a-5p	AMBRA1
hsa_circ_0022989	hsa-miR-198	hsa-miR-212-5p	hsa-miR-93-3p	hsa-miR-141-5p	hsa-miR-29a-5p	DPP3
hsa_circ_0023033	hsa-miR-520f-3p	hsa-miR-433-3p	hsa-miR-93-3p	hsa-miR-302d-3p	hsa-miR-181a-2-3p	C11orf80
hsa_circ_0023179	hsa-miR-770-5p	hsa-miR-588	hsa-miR-125a-3p	hsa-miR-557	hsa-miR-377-5p	LRP5
hsa_circ_0023397	hsa-miR-431-5p	hsa-miR-106b-3p	hsa-miR-125a-5p	hsa-miR-125b-5p	hsa-miR-572	NADSYN1
hsa_circ_0024758	hsa-miR-216a-5p	hsa-miR-595	hsa-miR-607	hsa-miR-545-3p	hsa-miR-483-5p	STT3A
hsa_circ_0025388	hsa-miR-653-5p	hsa-miR-509-3p	hsa-miR-584-3p	hsa-miR-370-3p	hsa-miR-93-3p	A2ML1
hsa_circ_0004313	hsa-miR-198	hsa-miR-765	hsa-miR-619-3p	hsa-miR-576-3p	hsa-miR-217	LETMD1
hsa_circ_0028502	hsa-miR-365b-5p	hsa-miR-185-3p	hsa-miR-214-3p	hsa-miR-487b-5p	hsa-miR-29a-5p	SLC24A6
hsa_circ_0008784	hsa-miR-603	hsa-miR-362-5p	hsa-miR-124-3p	hsa-miR-377-3p	hsa-miR-670-5p	TNFRSF19
hsa_circ_0007404	hsa-miR-139-3p	hsa-miR-876-5p	hsa-miR-641	hsa-miR-502-5p	hsa-miR-1-5p	TMCO3
hsa_circ_0031724	hsa-miR-769-5p	hsa-miR-211-5p	hsa-miR-204-5p	hsa-miR-520g-3p	hsa-miR-144-5p	SEC23A
hsa_circ_0034044	hsa-miR-199b-5p	hsa-let-7a-3p	hsa-miR-98-3p	hsa-let-7f-1-3p	hsa-miR-552-5p	HERC2P3
hsa_circ_0000673	hsa-miR-33a-3p	hsa-let-7f-2-3p	hsa-miR-25-5p	hsa-miR-203a-3p	hsa-miR-1185-2-3p	RSL1D1
hsa_circ_0005699	hsa-miR-512-5p	hsa-miR-553	hsa-miR-489-3p	hsa-miR-105-5p	hsa-miR-627-3p	C16orf62
hsa_circ_0003645	hsa-miR-1301-3p	hsa-miR-422a	hsa-miR-378a-3p	hsa-miR-602	hsa-miR-378d	C16orf62
hsa_circ_0038773	hsa-miR-365a-5p	hsa-miR-16-5p	hsa-miR-15a-3p	hsa-miR-515-5p	hsa-miR-627-5p	XPO6
hsa_circ_0000707	hsa-miR-136-5p	hsa-miR-145-5p	hsa-miR-370-3p	hsa-miR-26b-3p	hsa-miR-488-5p	CBFB
hsa_circ_0005615	hsa-miR-744-5p	hsa-miR-661	hsa-miR-9-5p	hsa-miR-377-5p	hsa-miR-205-5p	NFATC3
hsa_circ_0005016	hsa-miR-92a-2-5p	hsa-miR-449a	hsa-miR-449b-5p	hsa-miR-449c-5p	hsa-miR-608	IST1
hsa_circ_0041821	hsa-miR-18a-3p	hsa-miR-519a-5p	hsa-miR-519b-5p	hsa-miR-519c-5p	hsa-miR-518e-5p	NEURL4
hsa_circ_0041829	hsa-miR-34a-5p	hsa-miR-34c-5p	hsa-miR-128-1-5p	hsa-miR-449a	hsa-miR-449b-5p	NEURL4
hsa_circ_0042819	hsa-miR-302b-3p	hsa-miR-520c-3p	hsa-miR-520b	hsa-miR-302a-3p	hsa-miR-302c-3p	SSH2
hsa_circ_0005397	hsa-miR-26b-3p	hsa-miR-382-5p	hsa-miR-181a-3p	hsa-miR-330-5p	hsa-miR-125a-3p	RHOT1
hsa_circ_0043428	hsa-miR-143-5p	hsa-miR-544a	hsa-miR-105-5p	hsa-miR-765	hsa-miR-371a-5p	MED1
hsa_circ_0043691	hsa-miR-761	hsa-miR-450a-1-3p	hsa-miR-19a-5p	hsa-miR-580-3p	hsa-miR-544a	NT5C3L
hsa_circ_0005455	hsa-miR-573	hsa-miR-1264	hsa-miR-892b	hsa-miR-581	hsa-miR-146a-3p	KIAA1267
hsa_circ_0007591	hsa-miR-34a-3p	hsa-miR-564	hsa-miR-761	hsa-miR-502-3p	hsa-miR-432-3p	NPEPPS
hsa_circ_0044927	hsa-miR-410-5p	hsa-miR-584-5p	hsa-miR-148a-5p	hsa-miR-577	hsa-miR-494-5p	USP32
hsa_circ_0045737	hsa-miR-877-3p	hsa-miR-365a-5p	hsa-miR-512-5p	hsa-miR-128-1-5p	hsa-miR-128-3p	FBF1
hsa_circ_0045905	hsa-miR-520g-5p	hsa-miR-412-3p	hsa-miR-608	hsa-miR-597-3p	hsa-miR-127-5p	PGS1
hsa_circ_0006462	hsa-miR-141-5p	hsa-miR-887-5p	hsa-miR-136-5p	hsa-miR-671-5p	hsa-miR-216a-5p	NPLOC4
hsa_circ_0046290	hsa-miR-146a-3p	hsa-miR-185-5p	hsa-miR-593-5p	hsa-miR-657	hsa-miR-527	ASPSCR1
hsa_circ_0007144	hsa-miR-29b-2-5p	hsa-miR-29b-1-5p	hsa-miR-516b-5p	hsa-miR-888-3p	hsa-miR-659-3p	PTPRM

hsa_circ_0047159	hsa-miR-519c-3p	hsa-miR-608	hsa-miR-580-3p	hsa-miR-638	hsa-miR-494-5p	C18orf8
hsa_circ_0047303	hsa-miR-149-3p	hsa-miR-17-3p	hsa-miR-1301-3p	hsa-miR-509-5p	hsa-miR-516b-5p	ZNF521
hsa_circ_0007376	hsa-miR-571	hsa-miR-224-5p	hsa-miR-485-3p	hsa-miR-520f-3p	hsa-miR-342-3p	MAP2K2
hsa_circ_0003892	hsa-miR-608	hsa-miR-134-3p	hsa-miR-650	hsa-miR-874-5p	hsa-miR-589-5p	LDLR
hsa_circ_0049998	hsa-miR-608	hsa-miR-449b-3p	hsa-miR-761	hsa-miR-506-5p	hsa-miR-145-5p	PGLS
hsa_circ_0050834	hsa-miR-486-3p	hsa-miR-328-5p	hsa-miR-125a-3p	hsa-miR-296-5p	hsa-miR-873-5p	RYR1
hsa_circ_0051260	hsa-miR-1224-5p	hsa-miR-151a-5p	hsa-miR-377-5p	hsa-miR-432-3p	hsa-miR-133a-5p	GRIK5
hsa_circ_0054302	hsa-miR-122-5p	hsa-miR-1301-3p	hsa-miR-22-3p	hsa-miR-124-3p	hsa-miR-141-3p	THADA
hsa_circ_0055054	hsa-miR-141-5p	hsa-miR-298	hsa-miR-509-3p	hsa-miR-557	hsa-miR-548c-5p	GFPT1
hsa_circ_0055161	hsa-miR-502-5p	hsa-miR-764	hsa-miR-671-5p	hsa-miR-615-3p	hsa-miR-146b-3p	CYP26B1
hsa_circ_0004029	hsa-miR-29a-5p	hsa-miR-140-5p	hsa-miR-203a-5p	hsa-miR-668-5p	hsa-miR-302c-3p	UXS1
hsa_circ_0005227	hsa-miR-767-3p	hsa-miR-548b-5p	hsa-miR-573	hsa-miR-548d-5p	hsa-miR-548c-5p	TANK
hsa_circ_0004757	hsa-miR-9-5p	hsa-miR-1264	hsa-miR-218-2-3p	hsa-miR-342-5p	hsa-miR-203a-5p	SATB2
hsa_circ_0008365	hsa-miR-432-5p	hsa-miR-103a-2-5p	hsa-miR-605-3p	hsa-miR-130a-5p	hsa-miR-765	SERPINE2
hsa_circ_0058753	hsa-miR-214-5p	hsa-miR-1301-3p	hsa-miR-125a-5p	hsa-miR-125b-5p	hsa-miR-541-5p	INPP5D
hsa_circ_0003923	hsa-miR-593-3p	hsa-miR-342-5p	hsa-miR-501-5p	hsa-miR-515-5p	hsa-miR-887-5p	UBE2F
hsa_circ_0004339	hsa-miR-296-3p	hsa-miR-135a-5p	hsa-miR-135b-5p	hsa-miR-548d-5p	hsa-miR-570-5p	DONSON
hsa_circ_0061776	hsa-miR-503-5p	hsa-miR-182-5p	hsa-miR-450a-2-3p	hsa-miR-581	hsa-miR-455-3p	BACE2
hsa_circ_0008870	hsa-miR-597-3p	hsa-miR-616-3p	hsa-miR-335-3p	hsa-miR-185-3p	hsa-miR-29a-5p	MAPK1
hsa_circ_0002406	hsa-miR-150-3p	hsa-miR-764	hsa-miR-570-5p	hsa-miR-657	hsa-miR-646	TBC1D22A
hsa_circ_0001280	hsa-miR-9-3p	hsa-miR-519e-5p	hsa-miR-515-5p	hsa-miR-511-5p	hsa-miR-578	CLASP2
hsa_circ_0001292	hsa-miR-221-5p	hsa-miR-608	hsa-miR-214-3p	hsa-miR-500a-5p	hsa-miR-591	SCAP
hsa_circ_0002023	hsa-miR-512-3p	hsa-miR-552-3p	hsa-miR-494-5p	hsa-miR-134-5p	hsa-miR-493-3p	CDC25A
hsa_circ_0001305	hsa-miR-10b-3p	hsa-miR-141-5p	hsa-miR-200c-3p	hsa-miR-548a-3p	hsa-miR-631	RBM5
hsa_circ_0067029	hsa-miR-491-5p	hsa-miR-607	hsa-miR-373-5p	hsa-miR-92a-2-5p	hsa-miR-671-5p	PDIA5
hsa_circ_0001333	hsa-miR-500a-5p	hsa-miR-591	hsa-miR-7-5p	hsa-miR-614	hsa-miR-660-5p	ZNF148
hsa_circ_0068563	hsa-miR-520d-3p	hsa-miR-520a-3p	hsa-miR-302c-3p	hsa-miR-302a-3p	hsa-miR-302b-3p	XXYLT1
hsa_circ_0069559	hsa-miR-215-3p	hsa-miR-105-3p	hsa-miR-877-3p	hsa-miR-605-5p	hsa-miR-615-5p	APBB2
hsa_circ_0004780	hsa-miR-877-3p	hsa-miR-136-3p	hsa-miR-450b-3p	hsa-miR-373-5p	hsa-miR-138-5p	LRBA
hsa_circ_0003227	hsa-miR-200b-3p	hsa-miR-429	hsa-miR-200c-3p	hsa-miR-573	hsa-miR-539-5p	NUP155
hsa_circ_0072430	hsa-miR-26a-2-3p	hsa-miR-26a-1-3p	hsa-miR-152-5p	hsa-miR-20b-3p	hsa-miR-367-5p	PARP8
hsa_circ_0072758	hsa-miR-544a	hsa-miR-670-3p	hsa-miR-617	hsa-miR-493-5p	hsa-miR-575	CCNB1
hsa_circ_0073177	hsa-miR-17-3p	hsa-miR-16-5p	hsa-miR-541-5p	hsa-miR-15a-5p	hsa-miR-195-5p	MSH3
hsa_circ_0005540	hsa-miR-103a-3p	hsa-miR-107	hsa-miR-634	hsa-miR-29b-3p	hsa-miR-29a-3p	MCTP1
hsa_circ_0003528	hsa-miR-421	hsa-miR-181a-2-3p	hsa-miR-505-3p	hsa-miR-429	hsa-miR-380-5p	SEC24A
hsa_circ_0074816	hsa-miR-21-3p	hsa-miR-651-3p	hsa-miR-27a-3p	hsa-miR-138-1-3p	hsa-miR-539-5p	EBF1
hsa_circ_0006232	hsa-miR-370-3p	hsa-miR-367-5p	hsa-miR-133a-5p	hsa-miR-138-5p	hsa-miR-512-3p	TRERF1
hsa_circ_0077929	hsa-miR-335-3p	hsa-miR-587	hsa-miR-1185-2-3p	hsa-miR-105-5p	hsa-miR-1185-1-3p	AHI1
hsa_circ_0078150	hsa-miR-506-3p	hsa-miR-587	hsa-miR-29a-3p	hsa-miR-29b-3p	hsa-miR-29c-3p	STXBP5
hsa_circ_0078346	hsa-miR-92a-2-5p	hsa-miR-103a-2-5p	hsa-miR-412-3p	hsa-miR-802	hsa-miR-328-5p	IPCEF1
hsa_circ_0003958	hsa-miR-302b-3p	hsa-miR-107	hsa-miR-103a-3p	hsa-miR-33b-5p	hsa-miR-654-5p	HIBADH
hsa_circ_0080425	hsa-miR-24-3p	hsa-miR-608	hsa-miR-29a-5p	hsa-miR-382-5p	hsa-miR-493-5p	WBSCR17
hsa_circ_0082564	hsa-miR-612	hsa-miR-330-3p	hsa-miR-1296-5p	hsa-miR-298	hsa-miR-135b-3p	CREB3L2
hsa_circ_0002976	hsa-miR-608	hsa-miR-1224-5p	hsa-let-7c-5p	hsa-miR-582-3p	hsa-miR-26a-1-3p	UBAP2
hsa_circ_0003141	hsa-miR-34c-5p	hsa-miR-449a	hsa-miR-449b-5p	hsa-miR-449c-5p	hsa-miR-370-3p	UBAP2
hsa_circ_0087640	hsa-miR-136-5p	hsa-miR-103a-3p	hsa-miR-107	hsa-miR-581	hsa-miR-605-3p	CDC14B
hsa_circ_0087641	hsa-miR-136-5p	hsa-miR-103a-3p	hsa-miR-107	hsa-miR-581	hsa-miR-605-3p	CDC14B
hsa_circ_0003597	hsa-miR-16-5p	hsa-miR-135b-5p	hsa-miR-424-5p	hsa-miR-135a-5p	hsa-miR-15a-5p	HSDL2
hsa_circ_0088128	hsa-miR-199b-3p	hsa-miR-199a-3p	hsa-miR-122-5p	hsa-miR-570-3p	hsa-miR-574-5p	SLC31A1
hsa_circ_0088865	hsa-miR-149-5p	hsa-miR-9-5p	hsa-miR-29b-1-5p	hsa-miR-642a-5p	hsa-miR-608	SPTAN1
hsa_circ_0089153	hsa-miR-608	hsa-miR-185-3p	hsa-miR-653-3p	hsa-miR-377-5p	hsa-miR-298	NUP214
hsa_circ_0001917	hsa-miR-218-1-3p	hsa-miR-764	hsa-miR-338-3p	hsa-miR-96-5p	hsa-miR-300	CASK
hsa_circ_0006789	hsa-miR-146a-5p	hsa-miR-146b-5p	hsa-miR-329-5p	hsa-miR-30c-1-3p	hsa-miR-30c-2-3p	SLC25A43
hsa_circ_0091934	hsa-miR-128-2-5p	hsa-miR-128-1-5p	hsa-miR-197-3p	hsa-miR-20b-5p	hsa-miR-17-5p	FLNA
hsa_circ_0092275	hsa-miR-146a-3p	hsa-miR-541-5p	hsa-miR-105-5p	hsa-miR-335-3p	hsa-miR-378a-5p	RPS8
hsa_circ_0092374	hsa-miR-296-3p	hsa-miR-146b-3p	hsa-miR-181d-3p	hsa-miR-504-3p	hsa-miR-328-5p	GADD45A
hsa_circ_0092312	hsa-miR-576-5p	hsa-miR-29a-5p	hsa-miR-30a-3p	hsa-miR-30e-3p	hsa-miR-105-3p	ANKS3
hsa_circ_0092299	hsa-miR-665	hsa-miR-214-3p	hsa-miR-550a-5p	hsa-miR-550a-3-5p	hsa-miR-33b-3p	MCM5
hsa_circ_0000376	hsa-miR-153-5p	hsa-miR-619-5p	hsa-miR-29b-2-5p	hsa-miR-329-5p	hsa-miR-635	PRB4
hsa_circ_0000589	hsa-miR-1224-3p	hsa-miR-432-5p	hsa-miR-149-3p	hsa-miR-509-5p	hsa-miR-675-3p	KLF13
hsa_circ_0000775	hsa-miR-671-5p					KIF18B
hsa_circ_0001525	hsa-let-7i-5p	hsa-let-7g-5p	hsa-miR-619-5p	hsa-let-7f-5p	hsa-miR-98-5p	C5orf56
hsa_circ_0000866	hsa-miR-450b-5p	hsa-miR-210-5p	hsa-miR-19b-1-5p	hsa-miR-198	hsa-miR-525-5p	RPS15
hsa_circ_0001455	hsa-miR-95-5p	hsa-miR-155-5p	hsa-miR-526b-3p	hsa-miR-1271-3p	hsa-miR-519e-3p	MARCH1
hsa_circ_0000965	hsa-miR-619-5p	hsa-miR-130b-5p	hsa-miR-211-5p	hsa-miR-29b-2-5p	hsa-miR-665	C19orf18
hsa_circ_0000033	hsa-miR-378a-3p	hsa-miR-378d	hsa-miR-422a	hsa-miR-298	hsa-miR-328-5p	CEP85
hsa_circ_0012265	hsa-miR-138-5p	hsa-miR-296-3p	hsa-miR-627-3p	hsa-miR-214-3p	hsa-miR-150-3p	GPBP1L1
hsa_circ_0000091	hsa-miR-380-3p	hsa-miR-141-5p	hsa-miR-329-5p	hsa-miR-21-3p	hsa-miR-30c-5p	RPAP2
hsa_circ_0014624	hsa-miR-423-5p	hsa-miR-608	hsa-miR-296-3p	hsa-miR-877-3p	hsa-miR-185-5p	GON4L
hsa_circ_0005758	hsa-miR-377-5p	hsa-miR-508-5p	hsa-miR-552-3p	hsa-miR-452-5p	hsa-miR-891a-3p	KIAA0907

hsa_circ_0015240	hsa-miR-605-3p	hsa-miR-93-3p	hsa-miR-298	hsa-miR-367-5p	hsa-miR-376a-2-5p	PRRC2C
hsa_circ_0016201	hsa-miR-424-5p	hsa-miR-15a-5p	hsa-miR-10b-3p	hsa-miR-650	hsa-miR-15b-5p	DSTYK
hsa_circ_0017443	hsa-miR-892b	hsa-miR-138-5p	hsa-miR-329-5p	hsa-miR-488-5p	hsa-miR-671-5p	WDR37
hsa_circ_0008523	hsa-miR-550a-5p	hsa-miR-550a-3-5p	hsa-miR-584-3p	hsa-miR-431-5p	hsa-miR-133a-3p	DDX21
hsa_circ_0006649	hsa-miR-218-2-3p	hsa-miR-574-5p	hsa-miR-544a	hsa-miR-1224-3p	hsa-miR-19b-1-5p	DLG5
hsa_circ_0020080	hsa-miR-26b-3p	hsa-miR-222-5p	hsa-let-7e-5p	hsa-let-7a-5p	hsa-let-7c-5p	FAM160B1
hsa_circ_0008915	hsa-miR-146a-3p	hsa-miR-137	hsa-miR-335-3p	hsa-miR-29a-5p	hsa-miR-9-5p	ZRANB1
hsa_circ_0021506	hsa-miR-19b-2-5p	hsa-miR-19b-1-5p	hsa-miR-767-3p	hsa-miR-506-5p	hsa-miR-550a-3p	ANO5
hsa_circ_0022614	hsa-miR-15b-3p	hsa-miR-130b-5p	hsa-miR-412-3p	hsa-miR-526b-5p	hsa-miR-299-3p	MARK2
hsa_circ_0023255	hsa-miR-298					CPT1A
hsa_circ_0007767	hsa-miR-627-3p	hsa-miR-539-5p	hsa-let-7i-5p	hsa-miR-190b	hsa-miR-98-5p	ALG8
hsa_circ_0006374	hsa-miR-526b-5p	hsa-miR-29b-2-5p	hsa-miR-493-3p	hsa-miR-587	hsa-miR-764	ARCN1
hsa_circ_0000375	hsa-miR-150-5p	hsa-miR-670-3p	hsa-miR-1301-3p	hsa-miR-762	hsa-miR-185-3p	IFFO1
hsa_circ_0008932	hsa-miR-455-3p	hsa-miR-770-5p	hsa-miR-525-5p	hsa-miR-141-5p	hsa-miR-223-3p	LRP6
hsa_circ_0025822	hsa-miR-767-3p	hsa-miR-103a-2-5p	hsa-miR-520e	hsa-miR-526b-3p	hsa-miR-373-3p	DENND5B
hsa_circ_0004538	hsa-miR-612	hsa-miR-212-5p	hsa-miR-764	hsa-let-7f-2-3p	hsa-miR-660-3p	CSRNP2
hsa_circ_0026978	hsa-miR-18a-3p	hsa-miR-190a-5p	hsa-miR-382-5p	hsa-miR-450a-5p	hsa-miR-624-3p	CS
hsa_circ_0028899	hsa-miR-374a-3p	hsa-miR-124-5p	hsa-miR-181b-5p	hsa-miR-181d-5p	hsa-miR-125b-1-3p	RNF10
hsa_circ_0029377	hsa-miR-487b-3p	hsa-miR-584-3p	hsa-miR-591	hsa-miR-593-5p	hsa-miR-145-5p	AACS
hsa_circ_0004711	hsa-miR-541-5p	hsa-miR-876-5p	hsa-miR-29b-2-5p	hsa-miR-140-5p	hsa-miR-659-5p	KIAA0564
hsa_circ_0031027	hsa-miR-139-3p	hsa-miR-143-3p	hsa-miR-876-5p	hsa-miR-641	hsa-miR-619-3p	TMCO3
hsa_circ_0004781	hsa-miR-338-3p	hsa-miR-524-5p	hsa-miR-570-3p	hsa-miR-9-5p	hsa-miR-520d-3p	PCNX
hsa_circ_0005327	hsa-miR-324-5p	hsa-miR-330-3p	hsa-miR-889-5p	hsa-miR-127-3p	hsa-miR-320a	ANXA2
hsa_circ_0000676	hsa-let-7e-5p	hsa-miR-124-3p	hsa-miR-877-5p	hsa-miR-506-3p	hsa-let-7c-5p	ABCC1
hsa_circ_0038111	hsa-miR-634	hsa-miR-511-5p	hsa-miR-660-3p	hsa-miR-134-3p	hsa-miR-580-3p	ABCC1
hsa_circ_0043278	hsa-miR-103a-2-5	hsa-miR-455-3p	hsa-miR-302c-3p	hsa-miR-520d-3p	hsa-miR-302b-3p	TADA2A
hsa_circ_0006220	hsa-miR-302c-3p	hsa-miR-520d-3p	hsa-miR-302b-3p	hsa-miR-302d-3p	hsa-miR-197-5p	TADA2A
hsa_circ_0044226	hsa-miR-153-5p	hsa-miR-532-5p	hsa-miR-26a-1-3p	hsa-miR-373-5p	hsa-miR-503-3p	CDC27
hsa_circ_0045714	hsa-miR-449c-5p	hsa-miR-328-3p	hsa-miR-193b-3p	hsa-miR-658	hsa-miR-2113	UNK
hsa_circ_0000814	hsa-miR-511-5p	hsa-miR-764	hsa-miR-339-5p	hsa-miR-211-5p	hsa-miR-204-5p	NPLOC4
hsa_circ_0049220	hsa-miR-627-3p	hsa-miR-130b-5p	hsa-miR-1468-5p	hsa-miR-659-3p	hsa-miR-335-3p	DNMT1
hsa_circ_0049224	hsa-miR-876-3p	hsa-miR-450b-5p	hsa-miR-1468-5p	hsa-miR-1468-3p	hsa-miR-627-3p	DNMT1
hsa_circ_0000977	hsa-miR-452-5p	hsa-miR-874-3p	hsa-miR-218-1-3p	hsa-miR-767-5p	hsa-miR-218-2-3p	NOL10
hsa_circ_0008911	hsa-miR-146a-3p	hsa-let-7f-2-3p	hsa-miR-612	hsa-miR-1301-3p	hsa-miR-593-3p	ZNF512
hsa_circ_0054021	hsa-miR-150-3p	hsa-miR-22-3p	hsa-miR-627-3p	hsa-miR-133a-5p	hsa-miR-1301-3p	CRIM1
hsa_circ_0054033	hsa-miR-515-5p	hsa-miR-449c-3p	hsa-miR-519e-5p	hsa-miR-205-5p	hsa-miR-588	STRN
hsa_circ_0055630	hsa-miR-511-5p	hsa-miR-485-5p	hsa-miR-574-5p	hsa-miR-539-5p	hsa-miR-198	LOC285033
hsa_circ_0009020	hsa-let-7c-3p	hsa-miR-224-5p	hsa-miR-1271-3p	hsa-miR-335-3p	hsa-miR-27a-3p	SEPT10
hsa_circ_0057582	hsa-miR-152-5p	hsa-miR-96-3p	hsa-miR-380-5p	hsa-miR-365a-5p	hsa-miR-103a-3p	HECW2
hsa_circ_0058497	hsa-miR-103a-2-5	hsa-miR-22-5p	hsa-miR-26b-3p	hsa-miR-609	hsa-miR-525-5p	RHBDD1
hsa_circ_0005265	hsa-miR-103a-2-5	hsa-miR-152-5p	hsa-miR-582-3p	hsa-miR-26b-3p	hsa-miR-140-5p	PTPRA
hsa_circ_0002113	hsa-miR-130a-5p	hsa-miR-516b-5p	hsa-miR-518c-5p	hsa-miR-367-5p	hsa-miR-671-5p	IFNGR2
hsa_circ_0008252	hsa-miR-145-5p	hsa-miR-876-3p	hsa-miR-875-3p	hsa-miR-671-3p	hsa-miR-630	UBE2L3
hsa_circ_0008044	hsa-miR-656-5p	hsa-miR-30b-3p	hsa-miR-432-5p	hsa-miR-93-3p	hsa-miR-494-5p	EWSR1
hsa_circ_0064019	hsa-miR-650	hsa-miR-539-5p	hsa-miR-132-5p	hsa-miR-585-3p	hsa-miR-1-5p	RABL2B
hsa_circ_0064644	hsa-miR-330-5p	hsa-miR-15a-5p	hsa-miR-429	hsa-miR-424-5p	hsa-miR-200b-3p	RBMS3
hsa_circ_0065173	hsa-miR-627-3p	hsa-miR-544a	hsa-miR-130b-5p	hsa-miR-22-5p	hsa-miR-335-3p	SETD2
hsa_circ_0065964	hsa-miR-497-5p	hsa-miR-424-5p	hsa-miR-15b-5p	hsa-miR-15a-5p	hsa-miR-214-3p	ACY1
hsa_circ_0067492	hsa-miR-484	hsa-miR-212-5p	hsa-miR-331-3p	hsa-miR-891a-3p	hsa-miR-624-5p	IL20RB
hsa_circ_0005873	hsa-miR-544a	hsa-miR-624-3p	hsa-miR-141-3p	hsa-miR-758-5p	hsa-miR-92b-5p	LRCH3
hsa_circ_0070245	hsa-miR-622	hsa-miR-103a-2-5p	hsa-miR-766-5p	hsa-miR-103a-3p	hsa-miR-107	SEC31A
hsa_circ_0070421	hsa-miR-1264	hsa-miR-30c-2-3p	hsa-miR-30c-1-3p	hsa-miR-363-5p	hsa-miR-513a-3p	HERC5
hsa_circ_0007380	hsa-miR-450b-3p	hsa-miR-708-3p	hsa-miR-644a	sa-miR-103a-2-5	hsa-miR-10b-3p	NSUN2
hsa_circ_0072279	hsa-miR-665	hsa-miR-874-5p	hsa-miR-188-3p	hsa-miR-29b-2-5p	hsa-miR-1271-3p	NUP155
hsa_circ_0002512	hsa-miR-607	hsa-miR-589-3p	hsa-miR-605-5p	hsa-miR-550a-5p	hsa-miR-550a-3-5p	DEPDC1B
hsa_circ_0073930	hsa-miR-455-3p	hsa-miR-29b-2-5p	hsa-miR-9-5p	hsa-miR-216a-3p	hsa-miR-493-3p	VDAC1
hsa_circ_0074323	hsa-miR-493-5p	hsa-miR-524-5p	hsa-miR-498	hsa-miR-218-2-3p	hsa-miR-296-3p	DIAPH1
hsa_circ_0075410	hsa-miR-221-5p	hsa-miR-153-5p	hsa-miR-592	hsa-miR-597-5p	hsa-miR-190b	DUSP22
hsa_circ_0003738	hsa-miR-122-3p	hsa-miR-490-5p	hsa-miR-92a-2-5p	hsa-miR-562	hsa-miR-302c-3p	LRRC16A
hsa_circ_0004712	hsa-miR-455-3p	hsa-let-7g-5p	hsa-miR-876-3p	hsa-miR-661	hsa-miR-323a-5p	PDE7B
hsa_circ_0078223	hsa-miR-576-5p	hsa-miR-887-5p	hsa-miR-892a	hsa-miR-425-5p	hsa-miR-138-5p	LATS1
hsa_circ_0007317	hsa-miR-22-5p	hsa-miR-498	hsa-miR-193a-5p	hsa-miR-493-3p	hsa-miR-148a-3p	IPCEF1
hsa_circ_0078616	hsa-miR-563	hsa-miR-10b-3p	hsa-miR-892b	hsa-miR-1264	hsa-miR-130a-5p	MAP3K4
hsa_circ_0078619	hsa-miR-136-5p	hsa-miR-1264	hsa-miR-29a-5p	hsa-miR-520f-3p	hsa-miR-339-5p	MAP3K4
hsa_circ_0079440	hsa-miR-670-3p	hsa-miR-495-5p	hsa-miR-103a-2-5p	hsa-miR-623	hsa-miR-20a-3p	PHF14
hsa_circ_0079619	hsa-miR-541-5p	hsa-miR-486-3p	hsa-miR-888-3p	hsa-miR-140-5p	hsa-miR-623	MPP6
hsa_circ_0082314	hsa-miR-372-3p	hsa-miR-302d-3p	hsa-miR-373-3p	hsa-miR-302a-3p	hsa-miR-302c-3p	NRF1
hsa_circ_0008555	hsa-miR-670-3p	hsa-miR-33a-5p	hsa-miR-33b-5p	hsa-miR-204-3p	hsa-miR-148b-5p	VPS37A
hsa_circ_0008303	hsa-miR-665	hsa-miR-134-3p	hsa-miR-193a-5p	hsa-miR-422a	hsa-miR-491-3p	PTDSS1
hsa_circ_0004380	hsa-miR-34b-5p	hsa-miR-20a-3p	hsa-miR-217	hsa-miR-449c-5p	hsa-miR-770-5p	TRAPPC9

hsa_circ_0086686	hsa-miR-512-3p	hsa-miR-370-3p	hsa-miR-504-3p	hsa-miR-193a-3p	hsa-miR-193b-3p	UBAP2
hsa_circ_0087300	hsa-miR-185-3p	hsa-miR-762	hsa-miR-9-5p	hsa-miR-345-3p	hsa-miR-103a-2-5p	TLE4
hsa_circ_0087305	hsa-miR-892b	hsa-miR-762	hsa-miR-214-5p	hsa-miR-30b-3p	hsa-miR-29b-1-5p	TLE4
hsa_circ_0088494	hsa-miR-302d-3p	hsa-miR-103a-2-5p	hsa-miR-302a-3p	hsa-miR-302b-3p	hsa-miR-302c-3p	NEK6
hsa_circ_0008240	hsa-miR-627-3p	hsa-miR-762	hsa-miR-767-5p	hsa-miR-423-3p	hsa-miR-449b-5p	FAM125B
hsa_circ_0092282	hsa-miR-532-5p	hsa-miR-766-3p	hsa-miR-490-5p	hsa-miR-384	hsa-miR-30a-3p	SNHG12
hsa_circ_0092338	hsa-miR-324-5p	hsa-miR-588	hsa-miR-140-5p	hsa-miR-216a-3p	hsa-miR-299-3p	NINL
hsa_circ_0092276	hsa-miR-149-5p	hsa-miR-892b	hsa-miR-431-3p	hsa-miR-516a-3p	hsa-miR-516b-3p	NISCH
hsa_circ_0092341	hsa-miR-328-3p	hsa-miR-212-5p	hsa-miR-135b-5p	hsa-miR-548d-3p	hsa-miR-135a-5p	C6orf132
hsa_circ_0092310	hsa-miR-19a-5p	hsa-miR-328-3p	hsa-miR-497-3p	hsa-miR-1301-3p	hsa-miR-584-3p	TJAP1
hsa_circ_0092346	hsa-miR-766-3p	hsa-miR-671-5p	hsa-miR-524-5p	hsa-miR-155-5p	hsa-miR-198	RPS4X

Table S6. Nucleotide sequences used in this study

**Primer pairs for qPCR/RT-PCR**	5 ' ⟶ 3 '
hsa_circ_0043278, circTADA2A-E5/E6- F	TGTGCACCAAGACCAAGGAG
hsa_circ_0043278, circTADA2A-E5/E6- R	AGGAAAATCTGAAGTAGTGA
hsa_circ_0000977, circNOL10-F	TGCTTCTGACGGCCAATGAA
hsa_circ_0000977, circNOL10-R	AACAGGCCAACTCTGTTTCGA
hsa_circ_0006220, circTADA2A- E6-F	GCCATTCCATTTCACTGCAG
hsa_circ_0006220, circTADA2A-E6- R	AGGTTCAGCAGGGTAGAT
hsa_circ_0007380, circNSUN2- F	CGTGCGGCCTCATCATAAGG
hsa_circ_0007380, circNSUN2-R	TGACGACTAATATTTCCAGAT
hsa_circ_0004538, circCSRNP2- F	TTCTGCGTGAGCACCTGAAG
hsa_circ_0004538, circCSRNP2- R	GCTTCTGCCGCTTCAGGATG
hsa_circ_0008240, circFAM125B-F	CCAACCTTCCCAGGACCAGT
hsa_circ_0008240, circFAM125B-R	CTGTGCAACTACGTCATAGC
hsa_circ_0044226, circCDC27- F	AGAATTGGACAAAGCATTAG
hsa_circ_0044226, circCDC27-R	TGGTTGTGGAGCTGTCACTA
hsa_circ_0000676, circABCC1- F	CTAGCCATCCTGAGATCCAA
hsa_circ_0000676, circABCC1- R	GAGGACCGTGTTCTGAAAGCA
human-ACTB-F	CATGTACGTTGCTATCCAGGC
human-ACTB-R	CTCCTTAATGTCACGCACGAT
circTADA2A-E6-F（hRLuc）	CCGCTCGAGGCAGGATGTAGCCAATCAAAT
circTADA2A-E6-R（hRLuc）	ATAAGAATGCGGCCGCAGTGAAATGGAATGGCTGTGT
miR-203a-3p-F	ATGGTTCGTGGGGTGAAATGTTTAGG
miR-203a-3p-R	GTGCAGGGTCCGAGGT
U6-F	CTCGCTTCGGCAGCACA
U6-R	AACGCTTCACGAATTTGCGT

**siRNA**	splicing junction
hsa_circ_0006220 circTADA2A siRNA #1	CCAUUUCACUGCAGGAUGUdTdT
hsa_circ_0006220 circTADA2A siRNA #2	CACUGCAGGAUGUAGCCAAdTdT
hsa_circ_0006220 circTADA2A siRNA #3	UUCCAUUUCACUGCAGGAUdTdT
Control siRNA	UUCUCCGAACGUGUCACGUTT
SOCS3 siRNA	CCAAGAACCUGCGCAUCCAdTdT
Control miRNA	CAGUACUUUUGUGUAGUACAA
miR-203a-3p inhibitor	CUAGUGGUCCUAAACAUUUCAC
miR-203a-3p mimic	GUGAAAUGUUUAGGACCACUAG

Fish probe

Cy3-labeled circTADA2A-E6 probe	CATCCTGCAGTGAAATGGAATGGC
FITC-labeled miR-203a-3p probe	CTAGTGGTCCTAAACATTTCAC
