# Supplementary material for: circTADA2As suppress breast cancer progression and metastasis via targeting miR-203a-3p/SOCS3 axis
Source: Cell Death Dis. 2019 Feb 20;10(3):175. doi: 10.1038/s41419-019-1382-y (PMC6382814; doi:10.1038/s41419-019-1382-y)
Supplement: Supplementary file 4 — Supplementary file 3 Supplementary Figures [file 41419_2019_1382_MOESM4_ESM.pdf]

Supplementary Figure

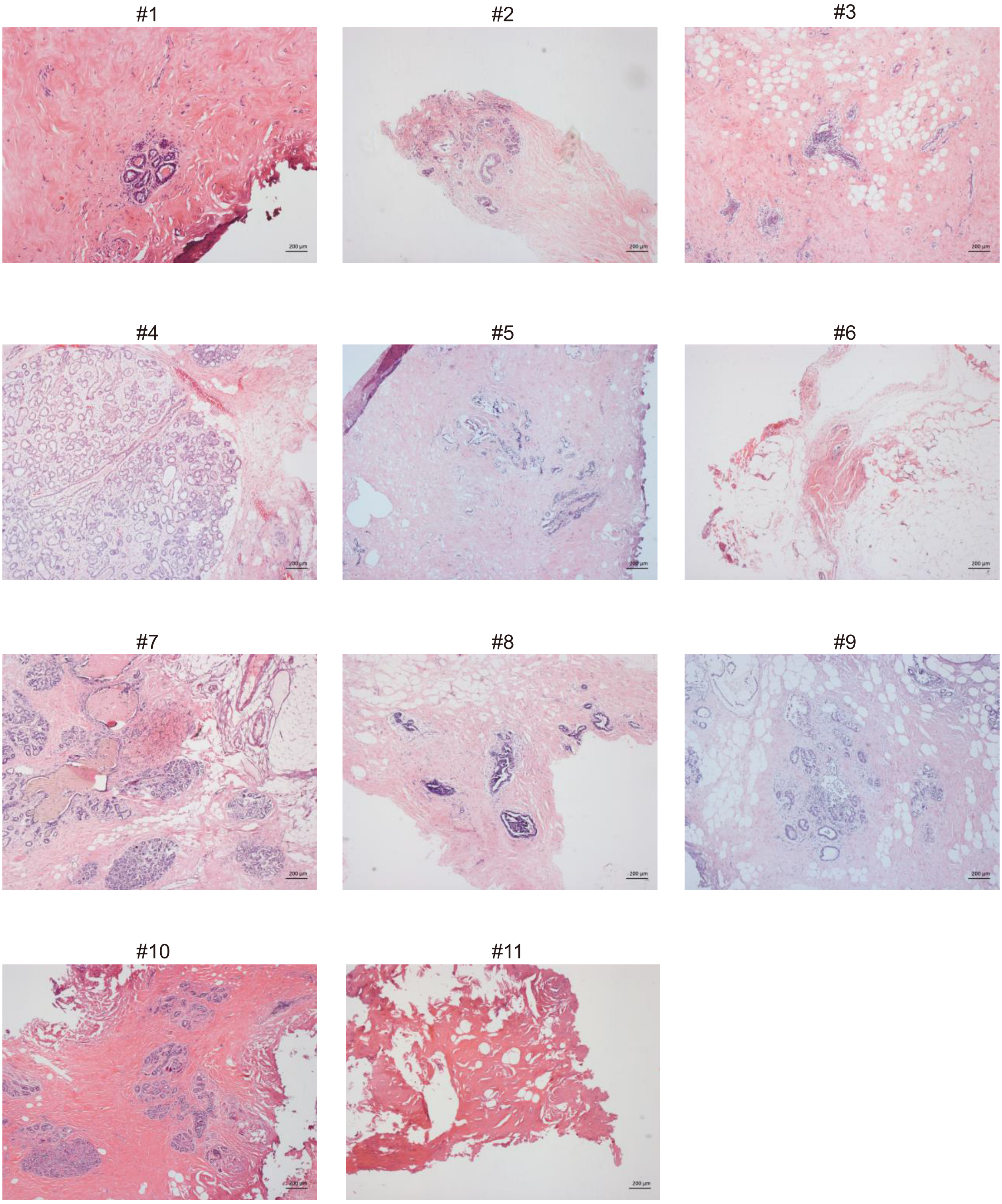

Figure S1. H & E staining of human normal mammary gland tissues (N = 11).

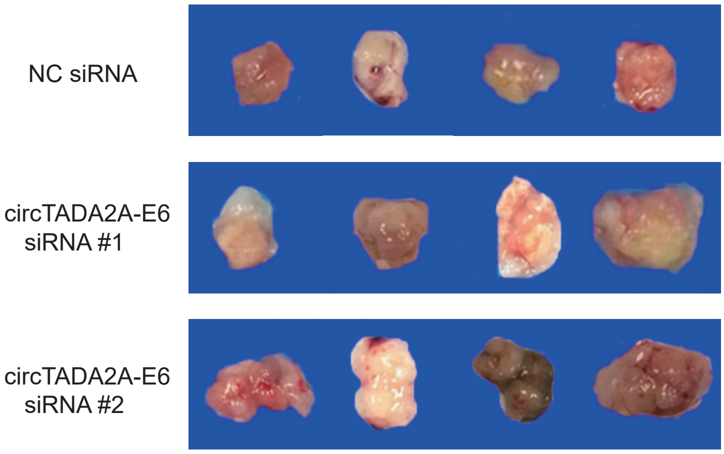

Figure S2. Images of isolated tumors.

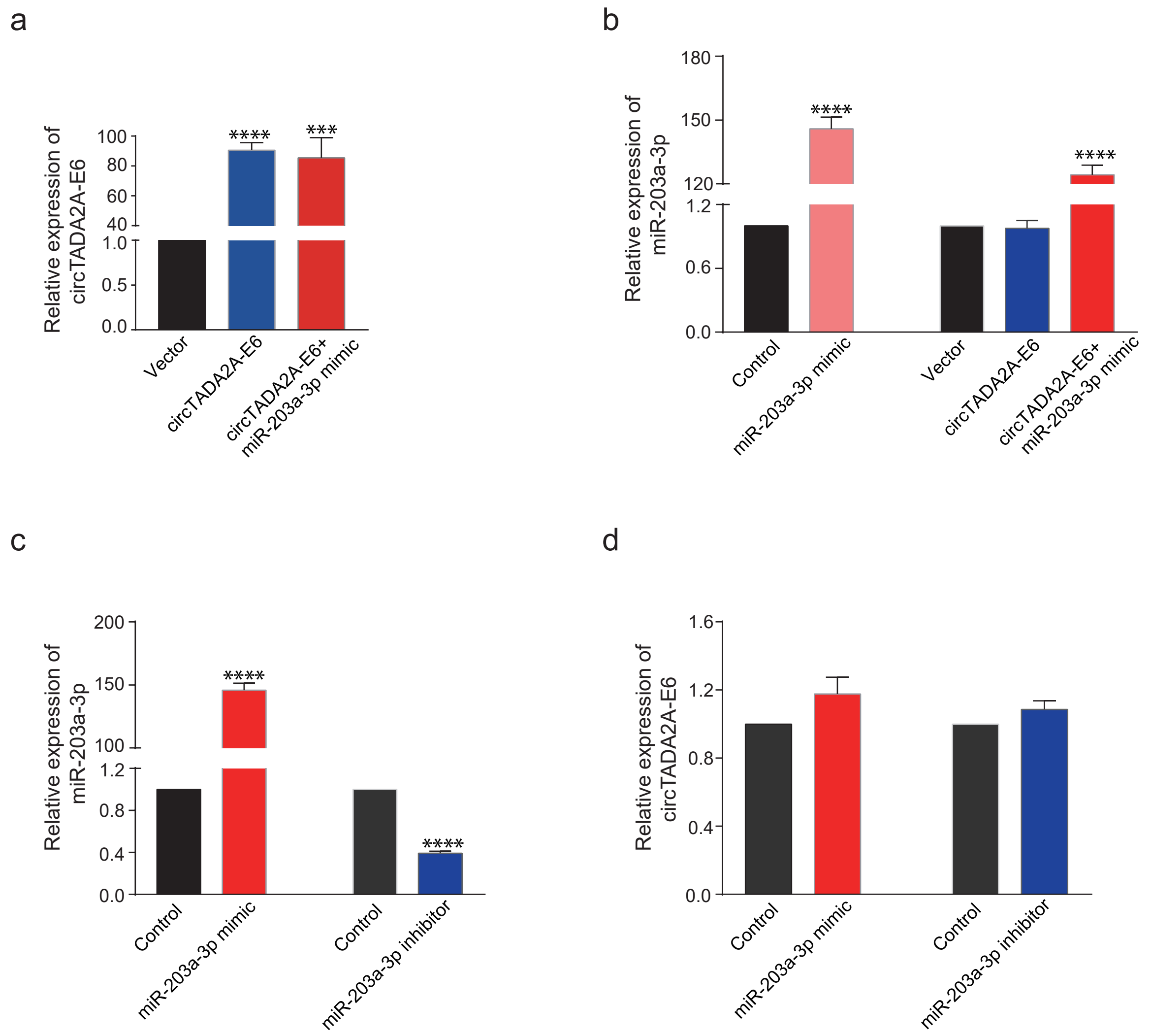

Figure S3. The relative expression of circTADA2A-E6 and miR-203a-3p.

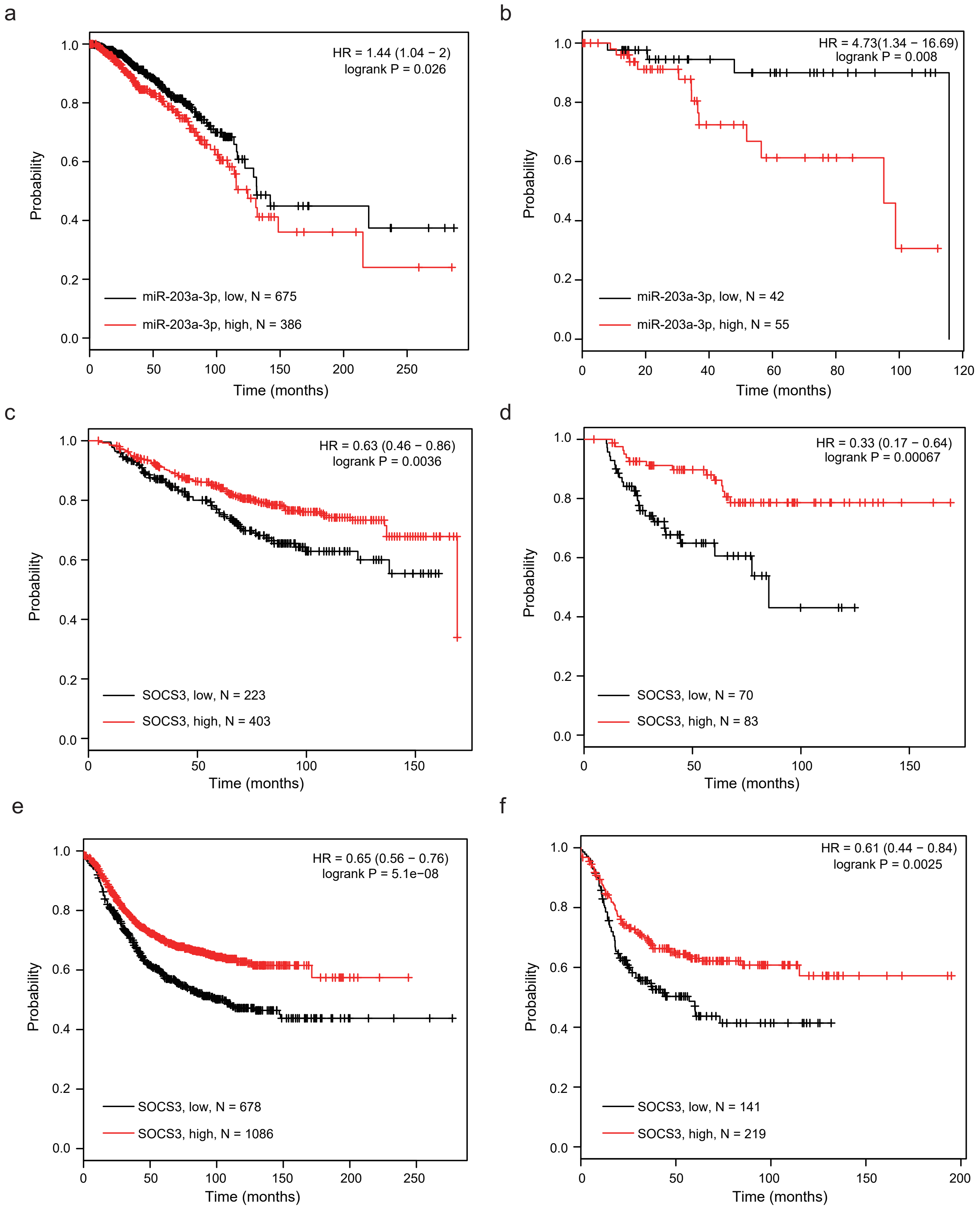

Figure S4. Kaplan–Meier survival curve analysis for the correlation between miR-203a-3p or SOCS3 expression and overall survival (OS) in all patients (a,c) or in TNBC(b,d). The correlation between SOCS3 expression and recurrence-free survival (RFS) in all patients(e) and in TNBC(f).

a

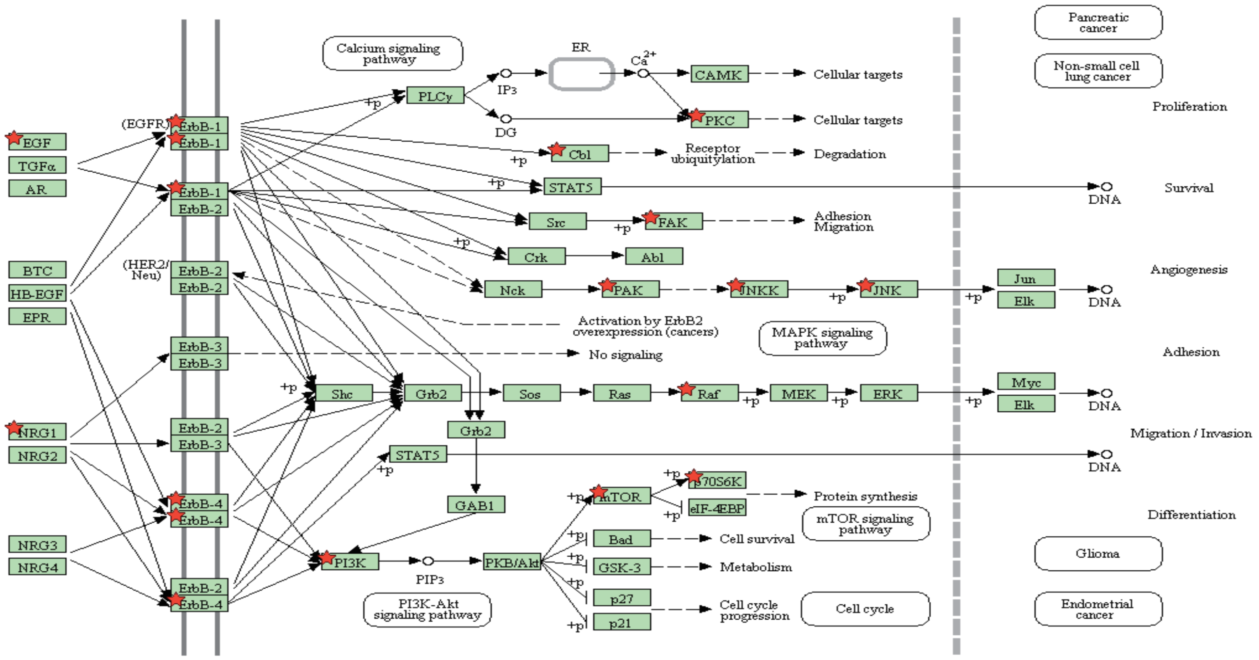

b

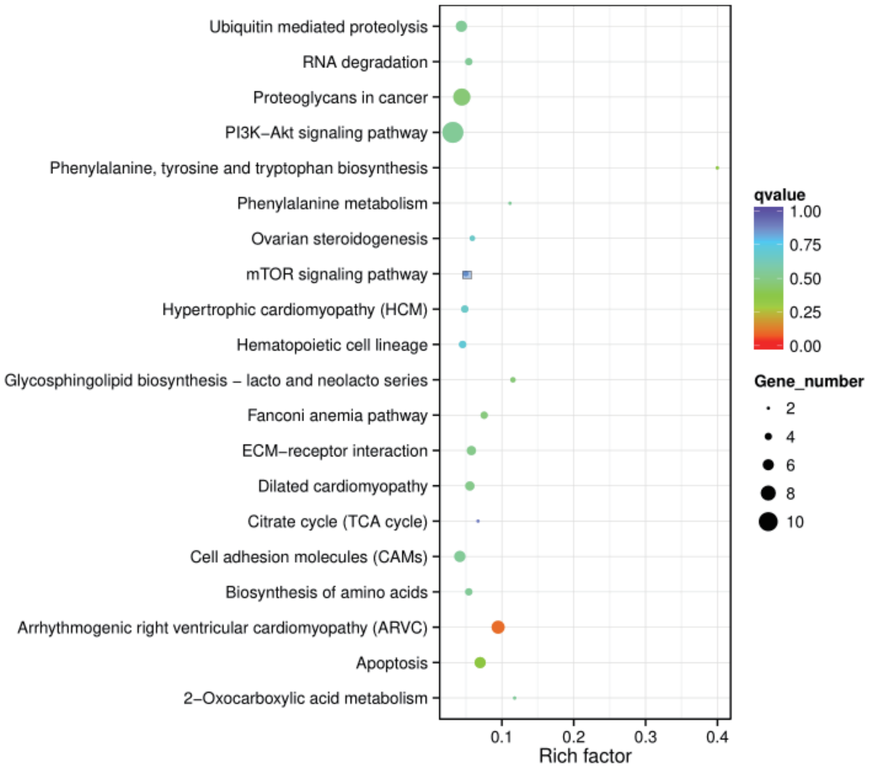

Figure S5. KEGG and GO analysis of circRNAs/miRNA/mRNA axis profiles.(a) The blinking showing the target genes could be regulated by circCDC27/miR-7 or miR-1276/mRNA axis. (b) Signaling pathway enriched by circTADA2A-E6/miR-203a-3p and miR-302c-3p/mRNA axis.

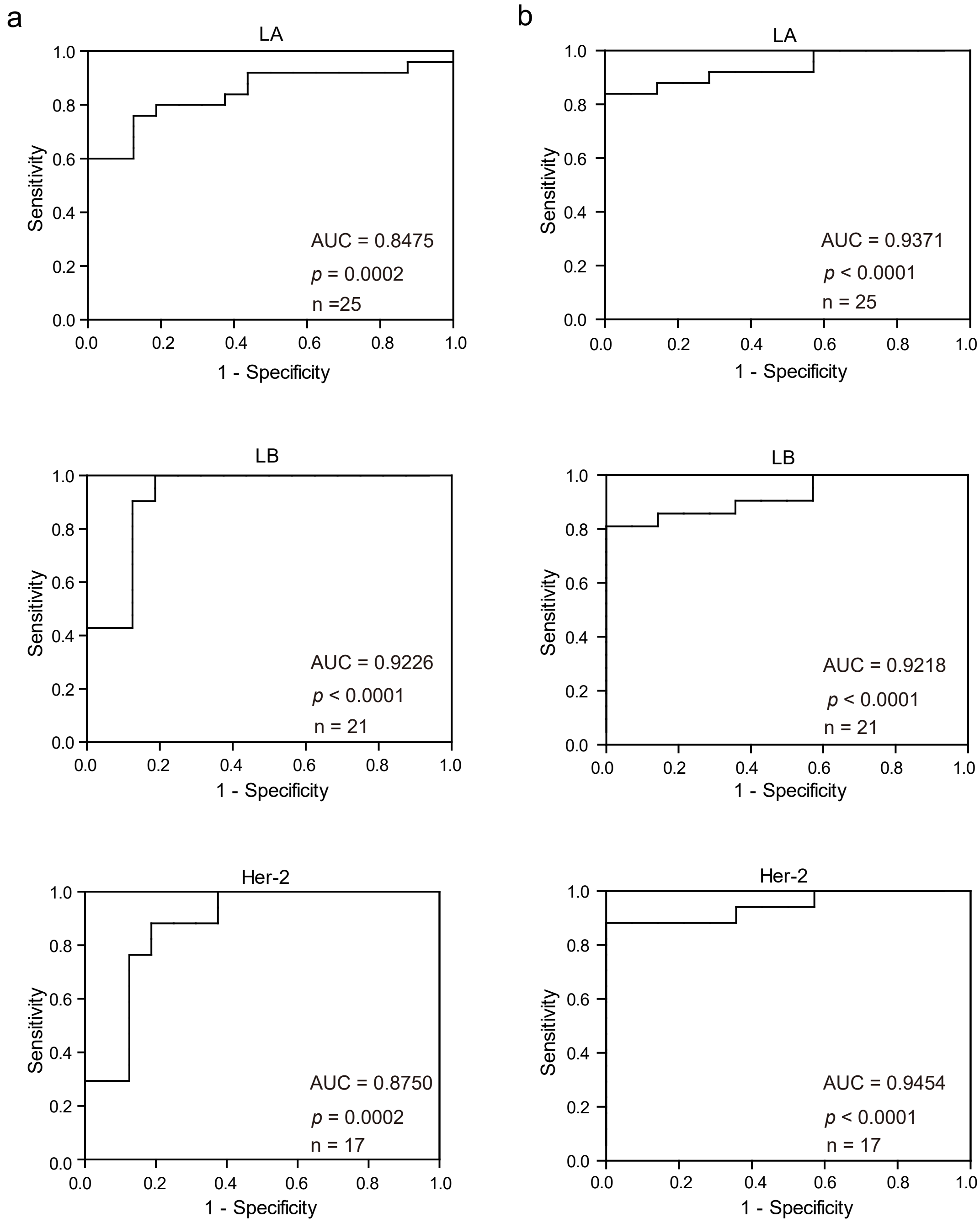

Figure S6. ROC analysis for circTADA2A-E6 (a) **and circTADA2A-E5/E6** (b) **in LA , LB and Her-2 breast cancer subtypes.**

a

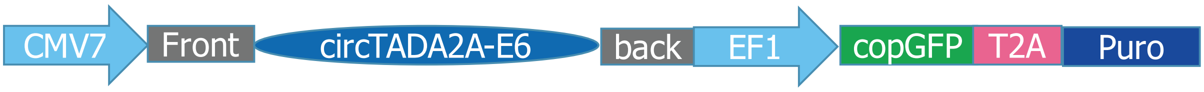

b

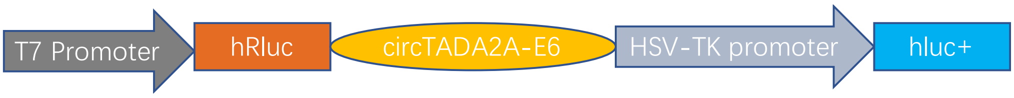

Figure S7. Schematic representation of the circTADA2A-E6 vectors.
(a) pLCDH-CMV-circTADA2A-EF1-copGFP-puro. (b) phRluc-circTADA2A-E6.

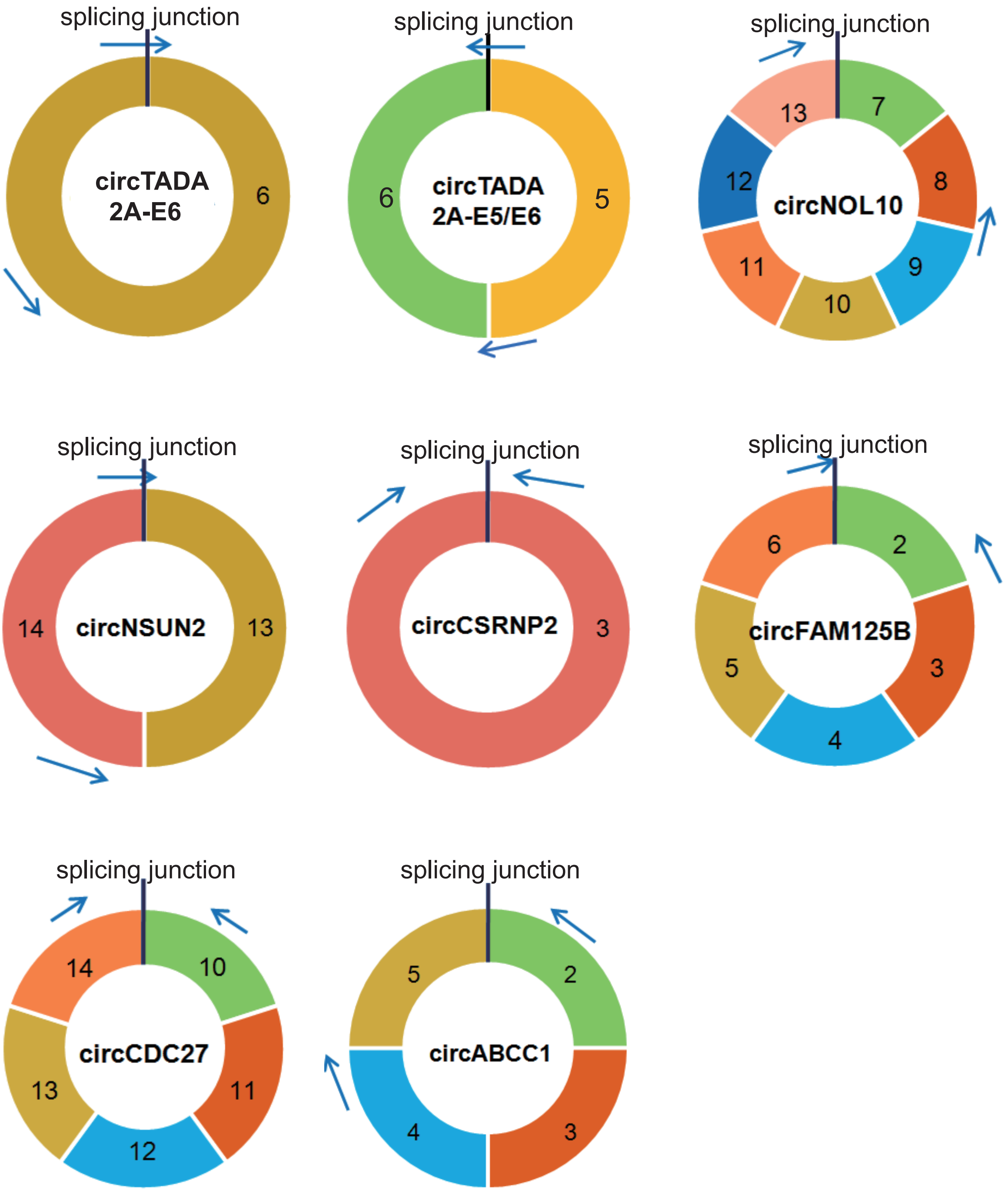

Figure S8. Schematic representation of the eight circRNAs formed by gene exon splicing. The arrows stand for divergent primers locus for qRT-PCR.

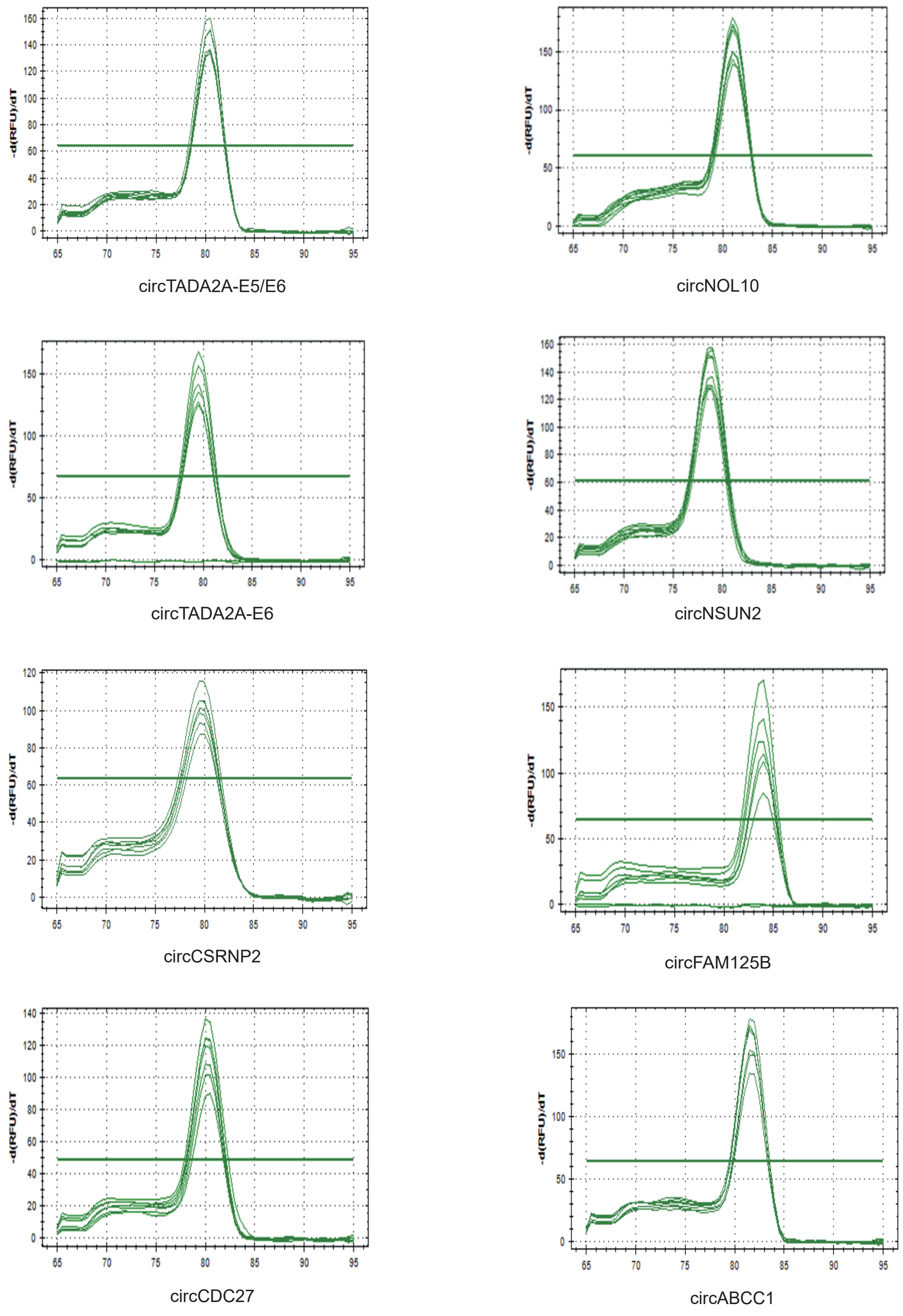

Figure S9. Melting curve of qRT-PCR showing a single, distinct product of the expected size for eight individual circRNAs.

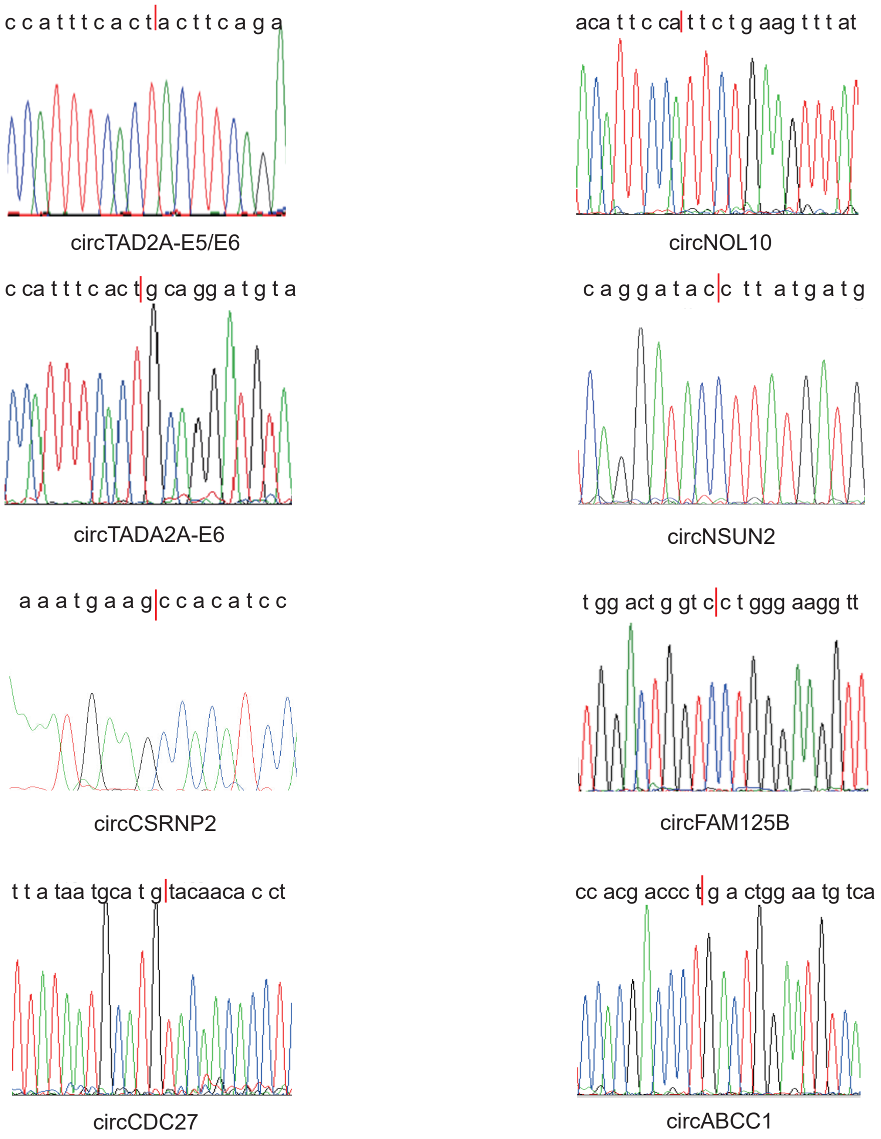

Figure S10. Sanger sequencing showing head-to-tail splicing for eight individual circRNAs. Sanger sequencing for qRT-PCR product confirmed the existing of a head-to-tail junction, the red lines indicated the backsplice junction.
